# Applying antibody-sensitive hypervariable region 1-deleted hepatitis C virus to the study of escape pathways of neutralizing human monoclonal antibody AR5A

**DOI:** 10.1371/journal.ppat.1006214

**Published:** 2017-02-23

**Authors:** Rodrigo Velázquez-Moctezuma, Mansun Law, Jens Bukh, Jannick Prentoe

**Affiliations:** 1 Copenhagen Hepatitis C Program (CO-HEP), Department of Infectious Diseases and Clinical Research Centre, Hvidovre Hospital and Department of Immunology and Microbiology, Faculty of Health and Medical Sciences, University of Copenhagen, Denmark; 2 Department of Immunology, The Scripps Research Institute, La Jolla, California, United States of America; Nationwide Children's Hospital, UNITED STATES

## Abstract

Hepatitis C virus (HCV) is a major cause of end-stage liver diseases. With 3–4 million new HCV infections yearly, a vaccine is urgently needed. A better understanding of virus escape from neutralizing antibodies and their corresponding epitopes are important for this effort. However, for viral isolates with high antibody resistance, or antibodies with moderate potency, it remains challenging to induce escape mutations in vitro. Here, as proof-of-concept, we used antibody-sensitive HVR1-deleted (ΔHVR1) viruses to generate escape mutants for a human monoclonal antibody, AR5A, targeting a rare cross-genotype conserved epitope. By analyzing the genotype 1a envelope proteins (E1/E2) of recovered Core-NS2 recombinant H77/JFH1_ΔHVR1_ and performing reverse genetic studies we found that resistance to AR5A was caused by substitution L665W, also conferring resistance to the parental H77/JFH1. The mutation did not induce viral fitness loss, but abrogated AR5A binding to HCV particles and intracellular E1/E2 complexes. Culturing J6/JFH1_ΔHVR1_ (genotype 2a), for which fitness was decreased by L665W, with AR5A generated AR5A-resistant viruses with the substitutions I345V, L665S, and S680T, which we introduced into J6/JFH1 and J6/JFH1_ΔHVR1_. I345V increased fitness but had no effect on AR5A resistance. L665S impaired fitness and decreased AR5A sensitivity, while S680T combined with L665S compensated for fitness loss and decreased AR5A sensitivity even further. Interestingly, S680T alone had no fitness effect but sensitized the virus to AR5A. Of note, H77/JFH1_L665S_ was non-viable. The resistance mutations did not affect cell-to-cell spread or E1/E2 interactions. Finally, introducing L665W, identified in genotype 1, into genotypes 2–6 parental and HVR1-deleted variants (not available for genotype 4a) we observed diverse effects on viral fitness and a universally pronounced reduction in AR5A sensitivity. Thus, we were able to take advantage of the neutralization-sensitive HVR1-deleted viruses to rapidly generate escape viruses aiding our understanding of the divergent escape pathways used by HCV to evade AR5A.

## Author summary

Worldwide hepatitis C virus (HCV) is one of the leading causes of chronic liver diseases, including cirrhosis and cancer. Treatment accessibility is limited and development of a preventive vaccine has proven difficult, partly due to the high mutation rate of the virus. Recent studies of HCV antibody neutralization resistance have revealed important information about escape pathways and barriers to escape for several clinically promising human monoclonal antibodies. However, due to the varying levels of antibody shielding between HCV isolates these studies have been mostly limited to a few neutralization-sensitive HCV isolates. Here, we took advantage of the fact that deletion of the hypervariable region 1 (HVR1) increased antibody sensitivity of HCV isolates by increasing the exposure of important epitopes, thus facilitating studies of antibody escape for neutralization resistant isolates. We identified escape mutations in the envelope glycoprotein E2, at amino acid position L665, which conferred antibody resistance in parental HCV viruses from genotypes 1–6. We found that antibody escape was associated with loss of binding to HCV particles and intracellular envelope protein complexes. We also identified escape substitutions at L665 that were isolate-specific. Thus, our data sheds new light on antibody resistance mechanisms across diverse HCV isolates.

## Introduction

About 150 million people are chronically infected with hepatitis C virus (HCV) with an increased risk of developing end-stage liver diseases, including cirrhosis and hepatocellular carcinoma [1–3]. Until recently, the treatment against HCV consisted of interferon and ribavirin but its efficacy was limited by side effects and a low rate of sustained virological response [4]. Progress in understanding HCV virology and the development of in vitro experimental systems to study antivirals has resulted in new interferon-free therapies [5]. These new treatment regimens consist of combinations of direct-acting antivirals (DAA) with or without ribavirin with greatly improved response rates. However, the high number of occult infections and the high cost of DAAs limit access, and the treatment does not provide protection against viral re-infection [2,6]. Thus, the need for a prophylactic HCV vaccine remains high.

The HCV genome encodes a single polyprotein that is processed into 3 structural proteins (Core, and envelope proteins E1 and E2), p7 and 6 nonstructural proteins (NS2-NS5B). HCV is an enveloped single positive-strand RNA virus belonging to the *Flaviviridae* family, and it is divided into seven major genotypes based on sequence homology [7,8]. The HCV envelope glycoprotein complex E1/E2, present on the surface of virus particles, plays a critical role in viral entry through interactions with cellular receptors such as CD81 [9], scavenger receptor class B type I (SR-BI) [10], the low-density lipoprotein receptor (LDLr) [11] and several late-stage host entry factors [12]. The E1/E2 complex is the target of neutralizing antibodies (NAbs) [13–17]. The presence of NAbs in the early phase of acute HCV infection has been associated with viral clearance [18,19]. Moreover, passive transfer of monoclonal and polyclonal antibodies has conferred protection against HCV infection in experimental animal models [20–25]. Efforts by several research groups have identified promising NAbs against HCV [26–33].

Using phage display libraries, human monoclonal antibodies (HMAbs) against conserved epitopes on E1 and E2 were isolated [27,33]. Epitope-masking allowed for isolation of antibodies against less frequently targeted epitopes [27], and one of these HMAbs, AR5A, which recognizes antigenic region 5, showed cross-genotype neutralization activity against cell cultured HCV [27]. Although the high mutation rate of HCV has been shown to enable the virus to escape neutralization [34–38], the barrier of escape for HMAb AR5A has not been assessed. The optimal vaccine candidate should induce NAbs against cross-genotype conserved epitopes with a universally high barrier to resistance.

Previously, antibody-specific resistance mutations have been induced by co-culturing HCV infected cells with NAbs or by direct neutralization of HCV virus followed by virus amplification of the non-neutralized quasispecies [39–43]. However, the variants studied has been limited to neutralization-sensitive isolates, such as JFH1 [40,41] or the recombinant virus HJ3-5 [43], introducing a potential bias.

Hypervariable regions of the structural glycoprotein E2 can influence neutralization sensitivity [44–46]. We have previously described increased susceptibility of HCV to antibody neutralization when the hypervariable region 1 (HVR1) of E2 is deleted [44,47]. The susceptibility to antibody neutralization for viruses without HVR1 has been shown to be between 10–1000 fold higher than for the parental virus [44,48]. HVR1 is not essential for infectivity given that viruses with this region deleted remained infectious in vitro [44;46] and in vivo [44,49,50]. Thus, we anticipated that the HVR1-deleted viruses could be an important tool for the study of antibody resistance, particularly for mapping conserved residues independent of this highly variable region frequently used by HCV to escape antibodies.

Here, we used highly sensitive HVR1-deleted variants to study AR5A resistance pathways for the two isolates H77 (genotype 1a) and J6 (genotype 2a) of medium or high antibody resistance, respectively; we also attempted escape studies for the original H77 and J6 viruses retaining HVR1. We induced AR5A-specific escape by culturing the JFH1-based Core-NS2 recombinants H77/JFH1_ΔHVR1_ and J6/JFH1_ΔHVR1_ in the presence of AR5A and identified mutations responsible for escape. Importantly, the mutations were also found to confer resistance in parental viruses. In addition, we were able to induce low-level non-AR5A specific resistance in H77/JFH1, but not J6/JFH1, by culturing these original viruses with high doses of AR5A. Finally, we used the JFH1-based Core-NS2 recombinant viruses of genotypes 1–6 to study resistance against AR5A. The information generated in these studies will be important to understanding HCV antibody escape, and the novel methodology using HVR1-deleted viruses could prove useful for designing future studies with other important NAbs against highly conserved epitopes. Furthermore, the present study provides novel insights into different pathways of AR5A virus escape for divergent isolates or even genotypes of this heterogeneous virus.

## Results

### Culturing of highly neutralization-sensitive HVR1-deleted H77/JFH1 with HMAb AR5A resulted in significantly delayed viral spread and selection of a resistant virus

**Fig 1:**
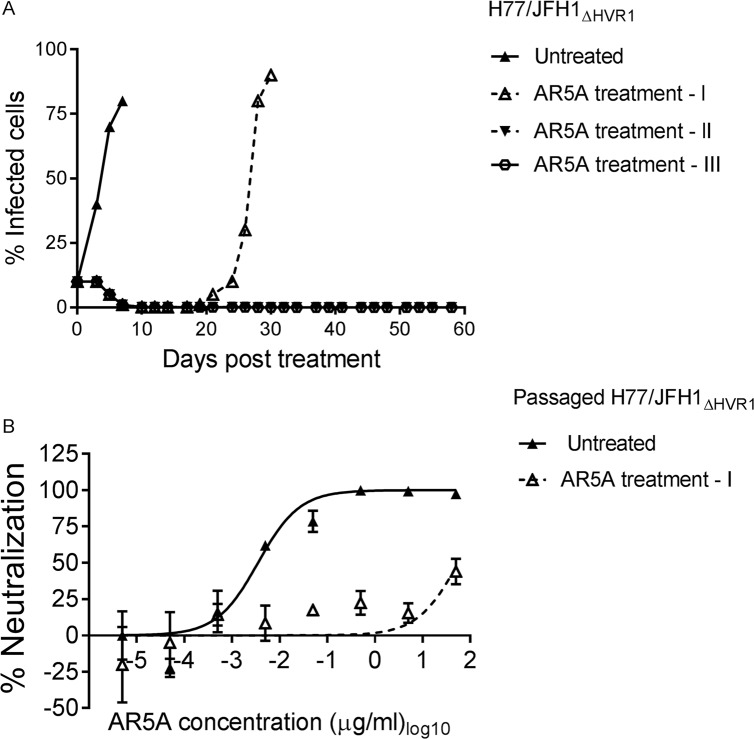
H77/JFH1_ΔHVR1_ escaped from HMAb AR5A antibody neutralization. (A) Huh7.5 cells were infected with H77/JFH1_ΔHVR1_ and treated with 5 μg/ml of AR5A antibody during the indicated period of time. Treatment I, II, III were treated independently and cultured along with the untreated control. (B) 1st passage of virus supernatant taken from treatment I and untreated control were subjected to a ten-fold dilution series of AR5A starting at 50 μg/ml. The virus/antibody mixes along with virus only were added to Huh7.5 cells for 4 hour prior to wash and addition of fresh medium. Following a total of 48 hour infection the cells were immunostained and the number of FFUs per well were counted as described in Materials and Methods. Neutralization data are shown as the mean of four replicates. Neutralization was related to infection of 8 virus replicates in the absence of antibody. Three-parameter curve-fitting was used to obtain sigmoidal dose-response curves. Error bars represents the standard errors of the mean.

On the assumption that the greatly increased sensitivity of HVR1-deleted HCV variants would make it easier to prevent spread when culturing the viruses in the presence of antibody, we attempted to generate neutralization escape mutants of HCV by culturing HVR1-deleted H77/JFH1 (H77/JFH1_ΔHVR1_) in Huh7.5 cells treated with relatively low doses of AR5A. Huh7.5 cells were infected with this genotype 1a recombinant at a multiplicity of infection (MOI) of 0.001. When the number of infected cells reached 10%, monitored by immuno-staining, cells were split into 4 wells and cultured either without antibody (untreated) or with 5 μg/ml of AR5A, which equals ~500 times the IC_50_ for H77/JFH1_ΔHVR1_ (treatments I, II, and III, Fig 1A). Seven days post-treatment the number of infected cells in the untreated well reached >80%, however, the number of infected cells in the treated wells decreased until it reached less than 1%. The percent of infected cells in treatment I increased from day 19 and reached >90% at day 30 post-initiation of antibody treatment. Treatment II and III were followed until day 58 when the cultures were closed due to infection suppresion as evidenced by the absence of antigen-positive cells for more than two weeks.

Culture supernatant of AR5A treated (day 30) and untreated virus (day 7) was used to generate 1^st^ passage virus stocks by infecting naive Huh7.5 cells. These stock viruses were tested in a dose-response focus forming units (FFU) reduction assay for AR5A antibody sensitivity (Fig 1B). The AR5A-treated virus was >10,000-fold more resistant against AR5A (Untreated virus IC_50_ = 0.0035 μg/ml; Treated virus IC_50_ > 50 μg/ml), indicating that the virus had escaped the HMAb. By direct sanger sequencing of the structural genes E1 and E2, we identified substitutions N417D (nucleotide change A1590G) and L665W (T2335G) in E2 (Table 1; all positions in the manuscript are given relative to H77 reference strain, GenBank accession number AF009606) in the AR5A treated virus; the untreated virus did not have any envelope mutations. The amino acid position N417 corresponds to glycosylation site 1 of E2. Thus, the resistant phenotype of the escape virus was likely due to one of these E2 substitutions.

**Table 1:** Coding mutations observed in the HCV envelope proteins. Mutations were found by direct sequence analysis of RT-PCR from recovered viruses. Red columns denote mutations observed to mediate low-level resistance against both AR3A and AR5A. Blue columns denote mutations shown to be involved in mediating specific high-level resistance against AR5A. *The mutation was dominant, but a minor a peak of the original nucleotide was observed. **The following amino acids were observed: HLYNRSIQTVPGK. §Data was taken from Los Alamos on 28th of December 2016. Of note, most isolates presented in the database are genotype 1, potentially skewing the results. -, No additional amino acids observed across genotypes.

HCV gene	E1	E1	E1	E1	E1	E2	E2	E2	E2	E2	E2	E2	E2
Nucleotide number and type													
Genome identity	**J6**	**J6**	**H77**	**S52**	**S52**	**J6**	**H77**	**S52**	**H77**	**S52**	**H77**	**J6**	**J6**
Genome position	**1124**	**1373**	**1386**	**1403**	**1455**	**1563**	**1589**	**1702**	**1940**	**2096**	**2334**	**2346**	**2390**
H77 abs. ref. (AF009606) position	**1125**	**1374**	**1387**	**1404**	**1456**	**1564**	**1590**	**1703**	**1941**	**2079**	**2335**	**2335**	**2379**
Nucleotide change	**A→C**	**A→G**	**C→A**	**A→T**	**C→T**	**G→A**	**A→G**	A→C*	**A→G**	**T→G**	**T→G**	**T→C**	**T→A**
**Amino Acid number and type**													
Polyprotein position	**262**	**345**	**349**	**355**	**372**	**408**	**417**	**454**	**534**	**586**	**665**	**669**	**684**
H77 abs. ref. (AF009606) position	**262**	**345**	**349**	**355**	**372**	**408**	**417**	**454**	**534**	**580**	**665**	**665**	**680**
Amino acid change	**I→L**	**I→V**	**A→D**	**I→F**	**A→V**	**R→K**	**N→D**	Q→H*	**T→A**	**F→V**	**L→W**	**L→S**	**S→T**
**Amino acids observed at least twice in Los Alamos database**^**§**^													
Within genotype	**IV**	**ILF**	**ATVG**	**IV**	**ATVGIF**	**RQKS**	**NSD**	**QE**	**TS**	**F**	**L**	**L**	**ST**
Across genotypes (unless also within genotypes)	**LM**	**VA**	**SFL**	**-**	**ML**	**NTAEH**	**-**	**ADGS**	**-**	**	**-**	**-**	**AV**

### L665W in HCV protein E2 conferred neutralization resistance against HMAb AR5A

**Fig 2:**
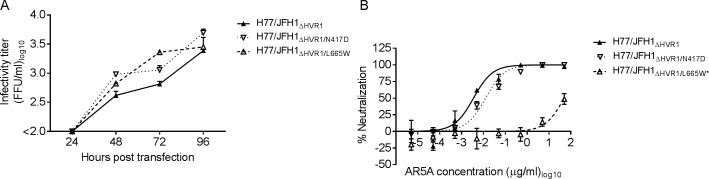
Substitution L665W in E2 of HCV increased resistance against antibody AR5A. (A) Huh7.5 cells were transfected with in vitro transcribed RNA of the indicated recombinants. Supernatants were collected and HCV infectivity titers were determined as indicated in Materials and Methods. (B) 1st passages of the indicated viruses were subjected to a ten-fold dilution series of AR5A starting at 50 μg/ml. The virus/antibody mixes along with virus only were added to Huh7.5 cells and after 48 hour infection the cells were immunostained and the number of FFUs per well were counted. Neutralization data are shown as the mean of four replicates normalized to 8 replicates of virus only. Three-parameter curve-fitting was used to obtain sigmoidal dose-response curves. Error bars represents the standard errors of the mean. *Virus harbored the substitution T534A (E2).

To determine which mutation conferred resistance against AR5A, mutations encoding N417D and L665W were introduced into H77/JFH1_ΔHVR1_. Viruses H77/JFH1_ΔHVR1_, H77/JFH1_ΔHVR1/N417D_ and H77/JFH1_ΔHVR1/L665W_ reached the peak of infection at day 4 post-transfection. Additionally, viruses harboring either N417D or L665W had titers similar to or slightly higher than the control virus H77/JFH1_ΔHVR1_ (Fig 2A). We generated a 1^st^ passage stock of all three viruses and their envelope gene sequences were analyzed. While we found no change for H77/JFH1_ΔHVR1_ and H77/JFH1_HVR1/N417D_, we observed the dominant E2 substitution T534A (A1941G, Table 1) for H77/JFH1_ΔHVR1/L665W_.

The neutralization sensitivity of these three viruses was compared using a dose-response FFU reduction assay for AR5A antibody sensitivity (Fig 2B). H77/JFH1_ΔHVR1/L665W_ (IC_50_ = 49 μg/ml) was highly resistant to antibody neutralization compared to H77/JFH1_ΔHVR1/N417D_ (IC_50_ = 0.012 μg/ml) and H77/JFH1_ΔHVR1_ (IC_50_ = 0.0035 μg/ml). To rule out that the substitution T534A, which appeared in the H77/JFH1_ΔHVR1/L665W_ virus, was involved in resistance against AR5A, we used a highly adapted HVR1-deleted virus, H77/JFH1_ΔHVR1/N417D/N532D_ for the investigation. We generated stocks of this virus with and without L665W. These virus stocks did not have any additional changes in the envelope genes and the virus harboring L665W was fully resistant to antibody neutralization using AR5A (S1 Fig). Thus, we showed that E2 substitution L665W alone conferred resistance to the HVR1-deleted 1a virus H77/JFH1 against AR5A.

### Substitution L665W in E2 conferred specific neutralization resistance against HMAb AR5A for parental H77/JFH1 virus retaining HVR1

**Fig 3:**
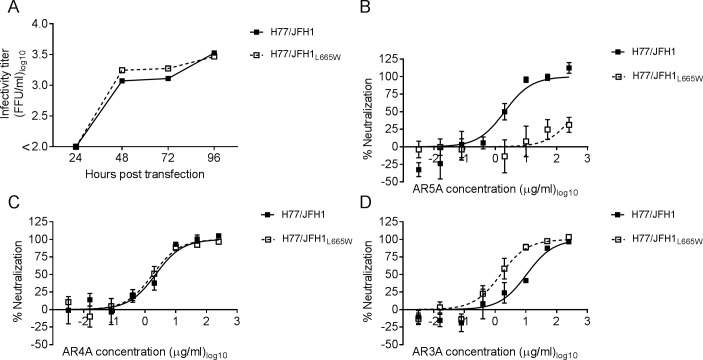
Parental virus H77/JFH1 with L665W was resistant against AR5A neutralization. (A) Huh7.5 cells were transfected with in vitro transcribed RNA of the indicated recombinants. Supernatants were collected as indicated and HCV infectivity titers were determined. (B-D) 1st passages of the indicated viruses were subjected to a five-fold dilution series of HMAb (B) AR5A, (C) AR4A, or (D) AR3A starting at 250 μg/ml. The virus/antibody mixtures along with virus only were added to Huh7.5 cells and after 48 hour infection the cells were immunostained and the number of FFUs per well were counted. Neutralization data are shown as the mean of 4 replicates with the standard error of the mean. Neutralization was related to infection of 8 virus replicates in the absence of antibody. Three-parameter curve-fitting was used to obtain sigmoidal dose-response curves. Error bars represent standard errors of the mean of 4 determinations.

To test if L665W conferred resistance against AR5A for the parental virus retaining HVR1 we constructed the recombinant H77/JFH1_L665W_. Following transfection of Huh7.5 cells with in vitro transcribed RNA, H77/JFH1 and H77/JFH1_L665W_ both reached titers of about 3.5 Log_10_ FFU/ml at day 4 post-transfection (Fig 3A). Virus supernatants were used to generate 1^st^ passage stocks and their envelope sequences were confirmed. Using these virus stocks we compared the susceptibility to AR5A using a dose-response FFU reduction assay (Fig 3B). H77/JFH1 was completely neutralized at the highest doses of antibody with an IC_50_ value of 1.9 μg/ml, whereas H77/JFH1_L665W_ was neutralized less than 50% even at the highest dose used in the assay (250 μg/ml).

Next, to test if the effects of L665W on H77/JFH1 sensitivity were specific for AR5A, we tested neutralization sensitivity against HMAbs AR3A and AR4A. These antibodies target conformational envelope epitopes that are distinct from the AR5A epitope [27,33]. The antibody susceptibility against AR4A was similar between the parental virus and H77/JFH1_L665W_ (Fig 3C). However, the susceptibility to AR3A was slightly higher for H77/JFH1_L665W_ (IC_50_ = 1.5 μg/ml) as compared with the parental virus (IC_50_ = 10 μg/ml) (Fig 3D), although this effect was not observed when we performed the analogous neutralization experiments for the HVR1-deleted virus H77/JFH1_ΔHVR1/L665W_ (S2 Fig). Thus, the mutation L665W conferred specific resistance against AR5A.

### L665W abrogated AR5A binding to the E1/E2 heterodimer, but had no effect on CD81, SR-BI and LDLr usage during entry

**Fig 4:**
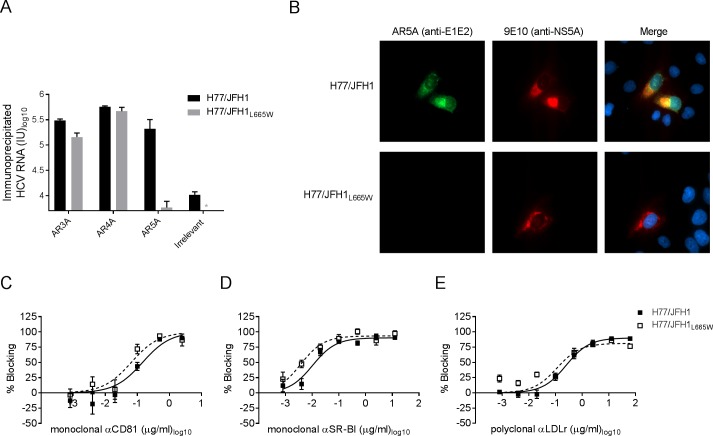
L665W decreased AR5A binding to H77/JFH1 particles and to the E1/E2 complex in H77/JFH1 infected cells, but did not alter receptor dependency. (A) Immunoprecipitation was carried out using anti-E1/E2 antibodies AR5A, AR4A, or the anti-E2 antibody, AR3A, or an irrelevant IgG as described in Material and Methods. RNA was measured in duplicates by RT-PCR (values given in international units, IU). The results represent the mean of the total amount of RNA in each sample. The error bar represents standard deviation. *HCV RNA titer below assay cut-off. (B) Huh7.5 cells were infected with virus H77/JFH1 or H77/JFH1_L665W_. After 48 hours cells were fixed and incubated with primary antibodies against NS5A (9E10) and E1/E2 (AR5A). Nuclei were counter-stained using Hoechst. Antibody binding was visualized using specific secondary antibodies coupled to fluorophores Alexa488 or Alexa594. Images were acquired using a Zeiss Axio Observer Z1. (C-E) Huh7.5 cells were incubated for 1 hour with dilution series of blocking antibodies against either (C) CD81, (D) SR-BI, or (E) LDLr or control antibody (see Material and Methods for specific antibodies). The indicated virus supernatants were added to Huh7.5 cells and incubated for 4 hour prior to wash and addition of fresh medium. Following a total of 48 hour infection the cells were immunostained and the number of FFUs per well were counted. Values are means of four replicates and normalized to 8 replicates of virus only. Three-parameter curve-fitting was used to obtain sigmoidal dose-response curves. Errors bars represent the standard errors of the mean.

To determine whether the decrease in neutralization sensitivity of H77/JFH1 harboring L665W was due to impaired binding of the antibody to the particle we performed immunoprecipitation of H77/JFH1 and H77/JFH1_L665W_ using HMAbs AR3A, AR4A, and AR5A. We quantified bead-associated HCV RNA by RT-qPCR and found greatly decreased immunoprecipitation of H77/JFH1_L665W_ by AR5A, whereas AR3A and AR4A immunoprecipitation was similar for the two viruses (Fig 4A). To determine whether AR5A binding was generally decreased by L665W, even outside the context of a mature virion, we carried out immunostaining of cells infected with viruses H77/JFH1 and H77/JFH1_L665W_ using AR5A and AR4A. We observed a strong AR5A-specific fluorescence signal for H77/JFH1 infected cells, but not for H77/JFH1_L665W_ infected cells (Fig 4B). In contrast, the antibody AR4A was able to recognize E2 for both viruses (S3 Fig).

Escape mutations in E2 have been shown to change protein structure and reduce binding to CD81 [51]. To investigate this for virus with L665W, we carried out dose-response entry blocking using antibodies against the receptors CD81 (Fig 4C), SR-BI (Fig 4D), and LDLr (Fig 4E). H77/JFH1_L665W_ was as susceptible to CD81, SR-BI, and LDLr blocking as the unmodified virus. Furthermore, H77/JFH1 and H77/JFH1_L665W_ virus entry were similarly reduced by incubation with soluble CD81 receptor prior to infection (S4 Fig).

### HVR1-deleted J6/JFH1 cultured in the presence of HMAb AR5A developed the envelope substitutions I345V, L665S, and S680T

**Fig 5:**
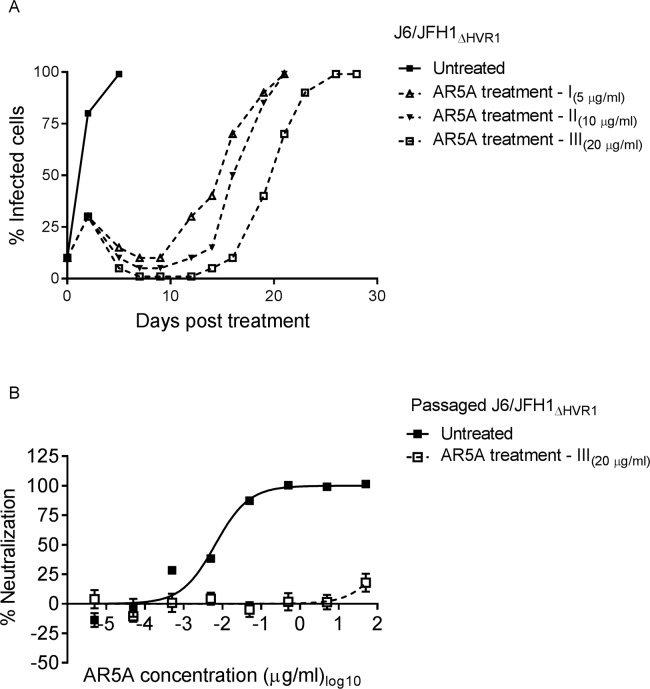
Culturing J6/JFH1_ΔHVR1_ with AR5A resulted in the selection of a resistant virus. (A) Huh7.5 cells were infected with virus J6/JFH1_ΔHVR1_ and treated with 20, 10, or 5 μg/ml of AR5A antibody during the indicated period of time. (B) 1st passage of virus supernatant from the 20 μg/ml treatment and the untreated control were subjected to a ten-fold dilution series of AR5A starting at 50 μg/ml. The virus/antibody mixes along with virus only were added to Huh7.5 cells and after 48 hour infection the cells were immunostained and the number of FFUs per well were counted. Neutralization data are shown as the mean of 4 replicates. Neutralization was related to infection in the absence of antibody. Three-parameter curve-fitting was used to obtain sigmoidal dose-response curves. Error bars represent the standard error of the mean.

As AR5A is cross-genotype-reactive we attempted to generate escape mutants for another HVR1-deleted virus, J6/JFH1_ΔHVR1_ (genotype 2a). Similar to the approach for H77/JFH1_ΔHVR1_ we infected Huh7.5 cells with J6/JFH1_ΔHVR1_ at an MOI of 0.001. After the infection reached 10%, cells were split into 4 wells and cultured without antibody or with antibody at concentrations of 5, 10, or 20 μg/ml of AR5A antibody (Fig 5A). While the control infection reached >90% infected cells at day 5 post-treatment, cells treated with 5, 10, and 20 μg/ml of AR5A reached >90% of infection only at day 19, 21, and 23, respectively. Sequences obtained from AR5A cultures treated with 5 and 10 μg/ml did not show any changes in the envelope proteins. However, the viral sequence from the supernatant harvested from the culture treated with 20 μg/ml displayed substitutions I345V (A1374G) in E1, and L665S (T2335C) and S680T (T2379A) in E2 (Table 1). The antibody susceptibility of the 1^st^ passage virus generated from the 20 μg/ml treatment was evaluated in a dose-response assay (Fig 5B). Similarly to what we observed for treated H77/JFH1_ΔHVR1_, the treated J6/JFH1_ΔHVR1_ virus (IC_50_ >50 μg/ml) was found to be >8000-fold less sensitive to AR5A compared to the parental J6/JFH1_ΔHVR1_ virus (IC_50_ = 0.006 μg/ml).

### Envelope substitutions L665S and S680T conferred AR5A resistance to J6/JFH1 both in the absence and presence of HVR1

**Fig 6:**
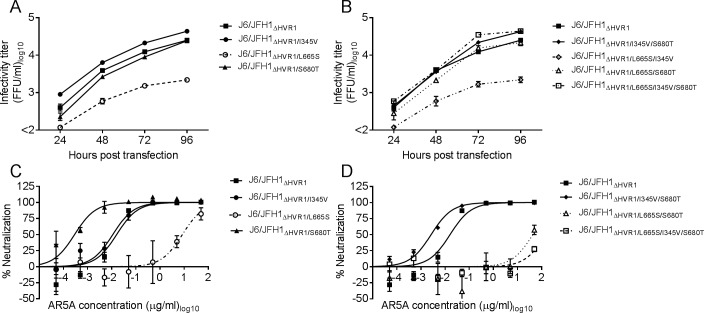
The substitutions L665S and S680T in E2 are both required to confer complete AR5A resistance to J6/JFH1_ΔHVR1_ without affecting virus fitness. Huh7.5 cells were transfected with in-vitro transcribed RNA of the recombinant viruses with (A) single or (B) combined substitutions I345V, L665S, and S680T. Supernatants were collected at the indicated time-points and HCV infectivity titers were determined. 1st passages of the viruses with (C) single or (D) combined substitutions I345V, L665S and S680T were subjected to a ten-fold dilution series of AR5A starting at 50 μg/ml. The virus/antibody mixes along with virus only were added to Huh7.5 cells and after 48 hour infection the cells were immunostained and the number of FFUs per well were counted as described in Materials and Methods. Neutralization values are the mean of four replicates and normalized to eight replicates of virus only. Three-parameter curve-fitting was used to obtain sigmoidal dose-response curves. Error bars represent the standard error of the mean.

Next, we introduced mutations encoding I345V, L665S, and S680T alone or in combination into J6/JFH1_ΔHVR1_. Viruses reached >90% of infection 4 days post-transfection. J6/JFH1_ΔHVR1_ harboring L665S or the combination L665S/I345V had greatly reduced fitness compared to the parental virus (Fig 6A and Fig 6B). In contrast, viruses containing S680T displayed titers similar to the parental virus even in combination with L665S and I345V, suggesting S680T was compensating for the deleterious effects of L665S. We attempted to generate a first passage stock for every mutant virus however J6/JFH1_ΔHVR1/L665S/I345V_ lost the L665S substitution. J6/JFH1_ΔHVR1/L665S_ (IC_50_ = 8.4 μg/ml) was >800-fold more resistant to AR5A than the parental virus (IC_50_ = 0.01 μg/ml) (Fig 6C), however it was still >5-fold more susceptible to neutralization than J6/JFH1_ΔHVR1/L665S/S680T_ (IC_50_ = 43 μg/ml) and J6/JFH1_ΔHVR1/L665S/I345V/S680T_ (IC_50_ >50 μg/ml) (Fig 6D), which were both similarly sensitive as the treated polyclonal escape virus (Fig 5B)_._ J6/JFH1_ΔHVR1/I345V_ had a neutralization profile similar to the parental virus (Fig 6C). Remarkably, J6/JFH1_ΔHVR1/S680T_ and J6/JFH1_ΔHVR1/I345V/S680T_ were more susceptible to neutralization with AR5A than J6/JFH1_ΔHVR1_.

**Fig 7:**
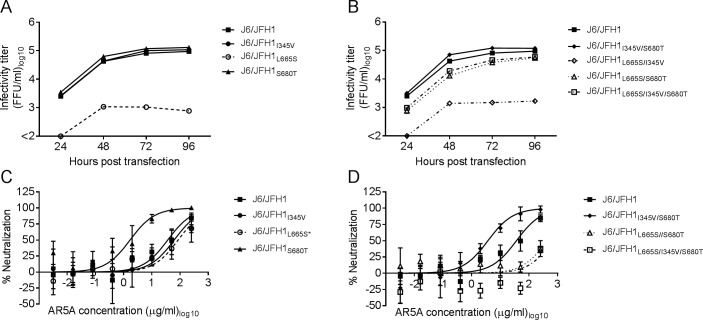
The substitution L665S combined with S680T conferred AR5A resistance to the parental recombinant virus J6/JFH1. Huh7.5 cells were transfected with in-vitro transcribed RNA of the indicated recombinant viruses with (A) single or (B) combined mutations. Supernatants were collected at the indicated time-points and HCV infectivity titers were calculated. 1st passages of the viruses with (C) single or (D) combined mutations were subjected to a five-fold dilution series of AR5A starting at 250 μg/ml. The virus/antibody mixes along with virus only were added to Huh7.5 cells and after 48 hour infection the cells were immunostained and the number of FFUs per well were counted. Neutralization data are shown as the mean of four replicates with the standard error of the mean. Neutralization was related to eight replicates of virus only. Three-parameter curve-fitting was used to obtain sigmoidal dose-response curves. Error bars represent standard errors of the mean. *Virus partially lost the substitution L665S.

Substitutions I345V, L665S, and S680T affected viability of parental J6/JFH1 in a similar way as described above for J6/JFH1_ΔHVR1_ (Fig 7A and Fig 7B). 1^st^ passage virus stocks were prepared for all the viruses, and again J6/JFH1_L665S/I345V_ lost the L665S mutation while J6/JFH1_L665S_ had a partial reversion of L665S. All other mutant viruses were generated successfully with no additional envelope mutations. The virus J6/JFH1_L665S_ did not appear resistant to AR5A, however, this could be due to the partial loss of the mutation. Viruses J6/JFH1_L665S/S680T_ (IC_50_ > 250μg/ml) and J6/JFH1_L665S/I345V/S680T_ (IC_50_ > 250 μg/ml) were >7-fold more resistant to antibody neutralization for AR5A than the parental virus (IC_50_ = 36 μg/ml) (Fig 7D). Similar to what we observed for the HVR1-deleted virus, the antibody susceptibility increased when viruses had the mutation S680T without L665S (Fig 7C and Fig 7D). Our results confirmed that the mutation L665S together with S680T conferred resistance to J6/JFH1 against AR5A.

### Changes in cell-to-cell spread not involved in AR5A resistance

To test whether L665W (for H77/JFH1_ΔHVR1_) or L665S and S680T (for J6/JFH1_ΔHVR1_) were influencing cell-to-cell spread we transfected Huh7.5 cells with HVR1-deleted HCV recombinants with and without these substitutions and mixed the transfected cells with naive cells either in the absence or in the presence of high levels of NAb, AR3A (to prevent cell-free spread). L665S decreased both cell-free and cell-to-cell spread (in line with our observations on the negative effect of this mutation on virus fitness, Fig 6A) whereas none of the other mutations had any effect on cell-to-cell spread (S5 Fig), indicating that changes in cell-to-cell spread were not the reason for the resistance of viruses harboring substitutions at residues 665 and 680.

### HCV was highly sensitive to most changes at the conserved E2 position L665, none of which altered the level of E1/E2 interaction

**Fig 8:**
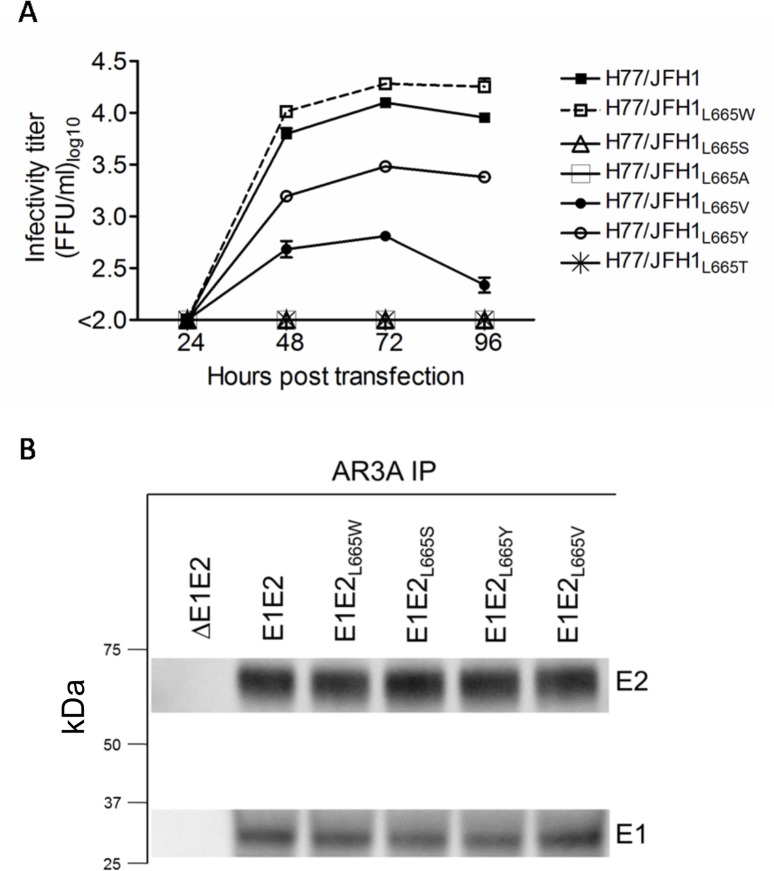
Position L665 in HCV E2 is highly sensitive to amino-acid substitutions. (A) The leucine at position 665 in H77/JFH1 was substituted for the indicated amino acids, and Huh7.5 cells were transfected with in vitro transcribed RNA of recombinants harboring these substitutions. Supernatants were collected at the indicated time-points and HCV infectivity titers were determined as described in Materials and Methods. (B) 293T cells were transiently transfected with vectors expressing L665 mutants of H77 E1E2 in cis. E1/E2 protein complexes were gently released using 1% nDDM detergent with protease inhibitors and benzonase. The samples were subjected to AR3A (conformational E2-specific antibody) immunoprecipitation and SDS-PAGE and western blot transfer was performed on the enriched fractions. E2 was detected using antibody H52 and E1 was detected using antibody A4.

Substitutions L665W and L665S decreased susceptibility against AR5A neutralization for viruses H77/JFH1 and J6/JFH1, respectively. To analyze the effects on viability and AR5A neutralization sensitivity of other amino acid substitutions we generated H77/JFH1 recombinants with serine (S), alanine (A), valine (V), tyrosine (Y), and threonine (T) at position 665. Viruses with substitutions L665Y and L665V were less infectious and the remaining substitutions rendered the virus non-viable resulting in the absence of infectious particles at the four tested time points post-transfection (Fig 8A). We attempted to generate 1^st^ passage virus stocks of H77/JFH1 harboring either L665Y or L665V, however infection with these viruses did not result in efficient viral spread and infected cells were not detected 3 weeks post-infection. Our data suggests that a very limited number of isolate-specific substitutions are allowed at this conserved E2 position. To address whether the decreased viability of the L665 mutants was due to perturbation of the E1/E2 interaction we performed immunoprecipitation (using the E2-conformational antibody, AR3A) of native E1/E2 complexes from 293T cells transiently expressing E1E2 in cis. Western blots of the E2-enriched fractions showed comparable levels of both E2 and the co-immunoprecipitated E1 protein (Fig 8B), strongly indicating that E1/E2 interaction was conserved for all L665 mutants.

### Culturing H77/JFH1 and J6/JFH1, retaining HVR1, in the presence of high doses of AR5A resulted in limited or no escape

**Fig 9:**
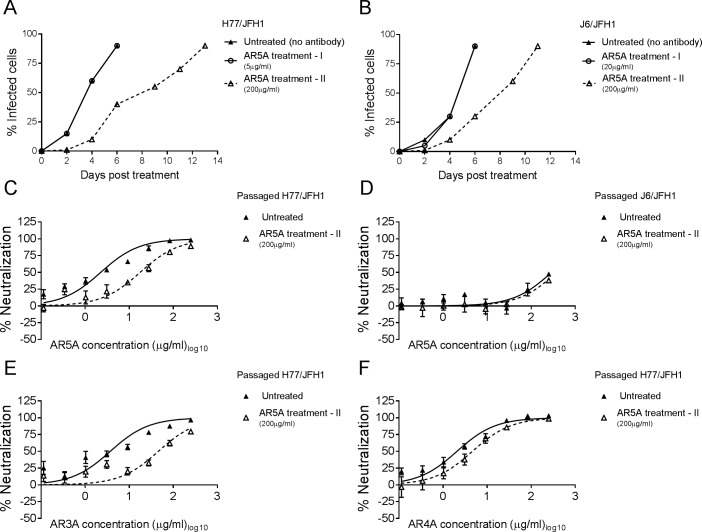
Culturing H77/JFH1 and J6/JFH1 with AR5A resulted in low or negligible escape. (A-B) Huh7.5 cells were infected with (A) H77/JFH1 and treated with 5 μg/ml (treatment I) or 200 μg/ml (treatment II) of AR5A or with (B) J6/JFH1 and treated with 20 μg/ml (treatment I) or 200 μg/ml (treatment II) of AR5A. Treatments were performed independently and cultured along with the untreated control. (C-D) 1st passage of virus supernatant taken from treatment II for both viruses and untreated control were subjected to a three-fold dilution series of AR5A, starting at 250 μg/ml. (E-F) The AR5A resistant H77/JFH1 virus from treatment II was subjected to neutralization with (E) AR3A and (F) AR4A as described above. The virus/antibody mixes along with virus only were added to Huh7.5 cells for 4 hour prior to wash and addition of fresh medium. Following a total of 48 hour infection the cells were immunostained and the number of FFUs per well were counted as described in Materials and Methods. Neutralization data are shown as the mean of four replicates. Neutralization was related to infection of 8 virus replicates in the absence of antibody. Three-parameter curve-fitting was used to obtain sigmoidal dose-response curves. Error bars represents the standard errors of the mean.

To evaluate whether substitutions at position 665 or 680 could be induced by culturing H77/JFH1 or J6/JFH1 in the presence of AR5A similar treatments as the ones described above were performed. Here, H77/JFH1 and J6/JFH1 were used to infect naive Huh7.5 cells at an MOI of 0.001. Once the virus spread to 1% of the cells both were cultured either without antibody or at the AR5A dose for which escape mutations were identified for the HVR1-deleted counterparts (5 μg/ml and 20 μg/ml, respectively), as well as a high dose of 200 μg/ml. Not surprisingly, only the high dose resulted in delayed viral spread (Fig 9A and 9B). Sequence analysis of the envelope protein encoding sequences from the treated cultures identified the unique coding substitution A349D (C1387A) for H77/JFH1 and R408K (G1564A) for J6/JFH1 in the samples treated at 200 μg/ml (Table 1).

These viruses were passaged to generate virus stocks with sequence-confirmed envelope proteins harboring these substitutions and without additional coding changes in the envelope proteins. In dose-response neutralization experiments against AR5A it was clear that the mutation A349D conferred low level resistance to H77/JFH1 (~8-fold, Fig 9C), whereas the mutation R408K had no effect on neutralization sensitivity of J6/JFH1 (Fig 9D). Interestingly, the mutation A349D also conferred low level resistance against AR3A (~12-fold, Fig 9E), but not AR4A (Fig 9F).

To test the effect of the mutations on virus viability we constructed the recombinants H77/JFH1_A349D_ and J6/JFH1_R408K_. Following transfection of Huh7.5 cells with in vitro transcribed RNA and infectivity titration of transfection supernatants it was evident that the mutation A349D increased the infectivity titer of H77/JFH1, whereas the mutation R408K had no effect on the infectivity of J6/JFH1 (S6A and S6B Fig). Thus, our data suggests that it is very difficult to induce specific escape mutations for resistant or even moderately resistant HCV recombinants as the virus is able to spread even at high antibody doses without acquiring specific resistance mutations.

### L665W conferred varied fitness cost across HCV genotypes 1–6, but universal AR5A resistance

**Fig 10:**
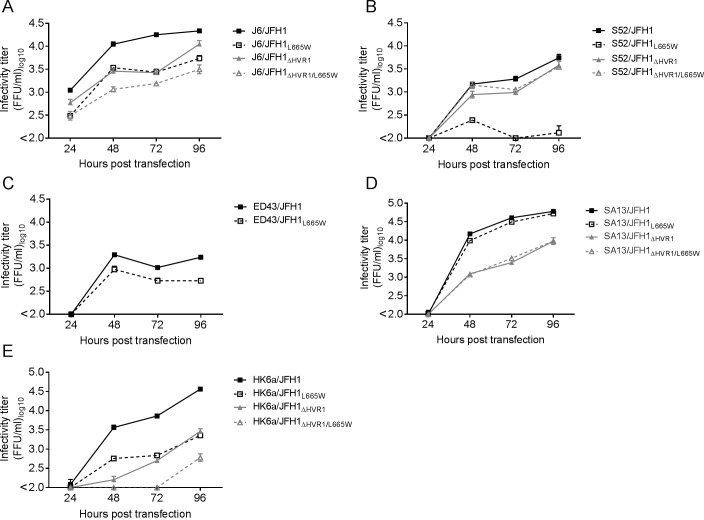
Differential fitness impact of the substitution L665W on prototype isolates of HCV genotypes 1–6. Mutations encoding L665W was introduced into cell-cultures adapted HCV Core-NS2 recombinants with or without HVR1 of genotypes (A) 2a (J6/JFH1), (B) 3a (S52/JFH1), (C) 4a (ED43/JFH1), (D) 5a (SA13/JFH1) and (E) 6a (HK6a/JFH1). Huh7.5 cells were transfected with in vitro transcribed RNA of the indicated recombinants. Supernatants were collected at the indicated time-point and HCV infectivity titers were determined as indicated in Materials and Methods.

Alignment of the E2 protein of the genotype 1a (H77), 2a (J6), 3a (S52), 4a (ED43), 5a (SA13), and 6a (HK6a) [52–54] revealed that the position L665 was conserved across these genotype 1–6 isolates, and indeed also universally conserved in the Los Alamos sequence database [55] (Table 1) and the European HCV database [56]. Since AR5A has demonstrated cross-genotype neutralizing potential [27,57], it was interesting to test whether changes at position L665 could confer AR5A-resistance in other HCV genotypes. Using the JFH1-based Core-NS2 HCV recombinants harboring envelope protein sequence from genotype 2a (J6), 3a (S52), 4a (ED43), 5a (SA13), and 6a (HK6a) [54;58–61] we introduced the mutations encoding L665W. The parental and the HVR1-deleted viruses with or without L665W were tested for all the genotypes except genotype 4 HVR1-deleted virus which was non-viable [44]. The effect of L665W on viral fitness varied greatly (Fig 10). While the parental and HVR1-deleted SA13/JFH1 viruses were virtually unaffected (Fig 10D), L665W seemed to attenuate both parental and HVR1-deleted J6/JFH1 (Fig 10A) and HK6a/JFH1 (Fig 10E), as well as the parental ED43/JFH1 (Fig 10C). On the other hand, L665W only affected the viability of the parental S52/JFH1 but not the HVR1-deleted counterpart (Fig 10B).

**Fig 11:**
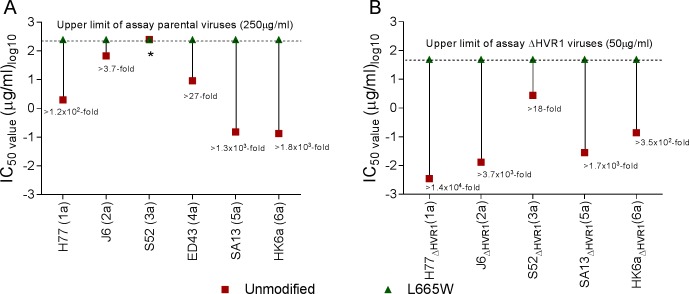
Substitution L665W increased AR5A resistance of HCV genotypes 1–6. 1st passage virus stocks of the indicated viruses were subjected to a dilution series of AR5A and the IC_50_ was determined for virus with (A) and without HVR1 (B). Numbers represent the number of times that the IC_50_ of a HCV virus with L665W is higher than its parental virus. J6/JFH1_L665W_ harbored the substitution I262L, S52/JFH1_L665W_ harbored the substitution I355F and S52/JFH1_ΔHVR1/L665W_ harbored the substitutions A372V, Q454H/q and F580V. *IC_50_ could not be determined as less than 50% neutralization was observed at the highest tested AR5A concentration.

We used supernatants from each transfection culture to infect Huh7.5 cells and generated 1^st^ passage stocks of each virus. The virus J6/JFH1_ΔHVR1/L665W_ developed the substitution I262L (A1125C, Table 1), the virus S52/JFH1_L665W_ developed I355F (A1404T, Table 1) and the virus S52/JFH1_ΔHVR1/L665W_ developed A372V (C1456T, Table 1), Q454H/q (A1703C/a, Table 1) and F580V (T2079G, Table 1). The remaining virus stocks did not acquire coding mutations in the envelope genes. Using these virus stocks we tested if the substitution L665W affected AR5A susceptibility across genotypes and found that it broadly decreased sensitivity to AR5A neutralization (Fig 11). The change in antibody susceptibility caused by L665W was higher for the HVR1-deleted viruses than for the parental viruses, most likely due to the generally increased neutralization sensitivity of the HVR1-deleted variants.

Finally, we compared neutralization sensitivity against the HMAb AR3A (S7 Fig) and AR4A (S8 Fig) for the different genotype viruses harboring L665W. With a few exceptions the neutralization susceptibility was unaffected by the introduction of L665W. J6/JFH1_ΔHVR1/L665W_ was 20-fold less susceptible to neutralization with AR3A; this was not the case for the parental virus J6/JFH1_L665W_ (S7A Fig). Virus ED43/JFH1_L665W_ was more susceptible to neutralization with both AR3A (S7C Fig) and AR4A (S8C Fig), in contrast both parental and HVR1-deleted SA13/JFH1 viruses were more resistant to antibody neutralization with AR3A (S7D Fig) and AR4A (S8D Fig). The other viruses had unaltered neutralization susceptibility against AR3A and AR4A. Thus, we concluded that overall L665W in E2 specifically reduced sensitivity against AR5A across HCV genotypes.

## Discussion

We identified different pathways of neutralization escape from the HMAb AR5A using the highly antibody susceptible HVR1-deleted viruses H77/JFH1_ΔHVR1_ and J6/JFH1_ΔHVR1_ and the original viruses H77/JFH1 and J6/JFH1. The mutation A349D, found by culturing H77/JFH1 with a high dose of AR5A, conferred broader low-level neutralization resistance for H77/JFH1. Mutations encoding amino acid changes at the highly conserved position L665, found by culturing neutralization sensitive HVR1-deleted H77/JFH1 and J6/JFH1 with at least 10-fold lower doses of AR5A, conferred specific high-level resistance against AR5A for both viruses, with and without HVR1, whereas mutation S680T was required to compensate for fitness loss as well as further increasing AR5A-specific resistance for J6/JFH1 and J6/JFH1_ΔHVR1_. In addition, we also observed several cases of adaptive mutations in the envelope proteins that likely also contributed to the ability of the virus to overcome AR5A neutralization. Finally, while we observed divergent, isolate-dependent, pathways to AR5A resistance we showed that substitutions at L665 could confer resistance across HCV genotypes 1–6, highlighting the potential importance of this position in AR5A resistance.

The development of an effective vaccine will be a cost-effective way to control HCV-associated liver disease worldwide [38;62;63]. In vitro studies of antibody resistance against HMAbs is important for HCV vaccine development to understand potential challenges with resistant viruses. Previous studies demonstrated an overlap between mutations found in resistant variants isolated in vitro and escape mutations identified from in vivo infection [39;40;42;43;64]. The relevance of in vitro escape studies has been shown for HMAb CBH-2, for which resistant variants had developed mutations at position D431 [40], also identified in a naturally occurring CBH-2 resistant variant [42]. Moreover, mutations at position N415 and N417 have been identified during in vitro escape studies against the antibody AP33 [39;43] and mutations at these same positions were also observed in resistant viruses found in liver transplant patients treated with HCV1, a HMAb recognizing the same E2 antigenic site [64]. Thus, in vitro escape studies can provide useful information on resistance pathways and barriers to resistance for virus NAb escape. Our previous findings that combinations of antibodies can have synergistic effects in neutralizing HCV [57], would be well complemented by detailed analyses concerning the barrier to resistance for these interesting epitopes.

The inherent broad neutralization antibody-resistance of some HCV isolates hinders the study of escape mutations for these viruses, partly due to the excessive amounts of antibody needed to suppress viral spread [65]. One way to minimize the use of HMAbs is to treat sensitive HCV recombinants such as the original JFH1, which is also not highly efficient in cell culture making it difficult for the virus to outgrow the antibody. Escape variants of JFH1 were isolated by culturing in the presence of around 10- or 50-times the IC_50_ concentrations of HMAbs CBH-2 or HC-11, respectively [40]. A distinct approach that reduced the amount of antibody relied on several iterations of a single neutralization step of the highly sensitive recombinant virus HJ3-5 with AP33 prior to inoculation of naive cells with the non-neutralized viruses [43]. The escaped variants were isolated by 3 rounds of antibody neutralization with >100 times the IC_50_ (100 μg/ml) of the antibody followed by amplification of the non-neutralized virus. However, the resistant viruses showed a considerable reduction of viral fitness indicating that the escaped virus needed additional mutations to compensate for the fitness loss. Another approach has been to passage the virus first in low doses of antibody followed by increasingly higher doses in subsequent passages, in the hope that escape will develop gradually under the low selective pressure. This approach has been shown to generate multiple JFH1 escape variants against HMAb HC33.1 [41]. While these are innovative solutions, they demand large amounts of antibody to generate escape variants for resistant HCV recombinants as shown in the case of selecting Jc1 (genotype 2a recombinant) escape mutants to AP33 [39]. Jc1 was serially passaged in the presence of AP33 at increasing concentrations up to ~40 times of the IC_90_ concentration (i.e. 200 μg/ml) [39].

There is currently no good way to induce escape mutations in naturally resistant HCV isolates (such as J6/JFH1) leading to a potential bias of the studied viruses and concomitantly the escape barriers. Here, we induced AR5A-specific escape in H77/JFH1_ΔHVR1_ and J6/JFH1_ΔHVR1_ at antibody doses (5 μg/ml and 20 μg/ml, respectively) that would be easily overcome by the parental viruses (Fig 9A and 9B). In fact, we were unable to induce escape mutations in J6/JFH1 even at 200 μg/ml (the highest dose we have observed others use to generate NAb escape [39]) and only induced low level (~8-fold) resistance at this high dose for H77/JFH1, which was not specific to AR5A. This highlights another advantage of using HVR1-deleted viruses to study specific NAb escape as it eliminates the acquisition of mutations in the envelope proteins that could contribute to a general increase in neutralization resistance thus impeding direct identification of antibody-specific escape mutations [66]. Here, we studied the moderately neutralization sensitive H77/JFH1 and the generally resistant J6/JFH1 (IC_50_ values against AR5A for H77/JFH1 and J6/JFH1 of 1.9 μg/ml and 36 μg/ml, respectively). The HVR1-deleted viruses had drastically reduced, and similar, IC_50_ values (IC_50_ was 0.0035 μg/ml for H77/JFH1_ΔHVR1_ and 0.0065 μg/ml for J6/JFH1_ΔHVR1_). Thus the deletion of HVR1 allowed us to induce escape at 5 μg/ml and 20 μg/ml of HMAb AR5A for H77/JFH1_ΔHVR1_ and J6/JFH1_ΔHVR1_, respectively. Interestingly, while H77/JFH1_ΔHVR1_ and J6/JFH1_ΔHVR1_ were similarly sensitive to neutralization with AR5A the latter was able to outgrow the antibody at 5 and 10 μg/ml. Although viral spread was significantly delayed, we did not observe escape variants following treatment of J6/JFH1_ΔHVR1_ with AR5A concentrations lower than 20 μg/ml, thus indicating that the highly infectious J6/JFH1_ΔHVR1_ was able to simply outgrow the antibody. Thus, it seems that viability of the virus used in escape studies is an important factor to consider when selecting the appropriate concentration of antibody. However, it should be noted that the viability of H77/JFH1 is similar to J6/JFH1_ΔHVR1_ indicating that decreased viability of HVR1-deleted viruses does not, by itself, explain why it is relatively easy to inhibit viral spread at low doses of antibody. In addition, using low multiplicities of the IC_50_ values of antibody others have isolated mutations that did not specifically affect virus susceptibility [39;41]. Thus, a high multiplicity of the IC_50_ values could be essential to the rapid isolation of resistant variants.

We propose that the use of the highly neutralization susceptible HVR1-deleted viruses allows for the isolation of antibody-specific resistant variants for otherwise neutralization-resistant HCV recombinants and eases the generation of escaped viruses for multiple isolates of different HCV genotypes. However, since the deletion of HVR1 has been shown to affect receptor interactions and the lipid composition of the viral particles [44;67], more studies using other NAbs are needed to evaluate if escape results obtained using the HVR1-deleted variants will have general relevance.

We identified escape mutations that arose in viruses cultured in the presence of AR5A. These mutations can be grouped according to their effects on virus susceptibility to AR5A and on virus viability. The only resistance mutation we identified through culturing viruses retaining HVR1 with AR5A was the substitution A349D, which was found by culturing H77/JFH1 at a high dose of AR5A. The mutation also conferred low level resistance against AR3A and could be similar in this respect to envelope mutations that broadly modulate NAb resistance such as those described by others [68–70]. However, it should be noted that aspartic acid (D) was not observed at this position in the Los Alamos database (Table 1), indicating that it is not a natural polymorphism. We identified two different mutations at position L665 (L665W for H77/JFH1 and L665S for J6/JFH1). Previously, we mapped the AR5A binding site to position R635 with an alanine substitution resulting in a reduction of relative binding of AR5A by 50–75% [27]. In the same study the alanine substitution at position L665 decreased the relative binding level of AR5A by 60%. However, since alanine is smaller than leucine and both amino acids are non-polar, the alanine scanning could be underestimating the importance of position L665 in AR5A binding. In contrast, the bigger amino acid group of tryptophan and the polarity of serine could induce the conformational change necessary to impair antibody binding.

We showed that L665W abrogated AR5A binding to HCV particles and to intracellular E1/E2 protein, while also greatly increasing resistance to AR5A neutralization for genotype 1–6 viruses. It should be noted that while we are not able to provide evidence for whether the different pathways to resistance against AR5A are isolate or genotype dependent the high envelope protein heterogeneity between the genotype isolates we have tested for AR5A resistance does represent the high diversity of HCV. Recently, we showed that polymorphisms at positions 636 and 708 could be involved in the higher resistance against HMAb AR5A of the HCV strain S52 [48], however the effect in resistance was lower than the one induced by the mutation L665W in the same genotype. Thus, our data strongly indicates that L665 is a part of the AR5A epitope. L665 was universally conserved in E2 sequences deposited in the Los Alamos HCV database [55] and the European HCV database [56] (Table 1), and the decrease in virus fitness for isolates from genotypes 2, 3, 4, and 6 with L665W suggests that L665 is important for virus infectivity. It has previously been shown that highly conserved epitopes tend to have a higher barrier to resistance, probably due to their importance in critical interactions [28;40]. However, we found that while L665 was highly conserved the barrier to resistance was isolate-dependent. The observed differences in the escape mutations for viruses with and without HVR1 suggests that it might be of interest in future studies to attempt to induce escape for viruses with and without HVR1 and with non-viral neutralization epitopes in their envelope proteins, such as the flag-epitope with neutralizing potential at the N-terminus of E2 [71], to investigate how escape mutations for such epitopes would cluster.

L665 is localized within the stem region that connects the E2 ectodomain to the C-terminal transmembrane region [72]. We previously proposed that the stem region could play a role in virus entry [73], however, E2 protein with the stem and transmembrane regions deleted could still interact with host entry factors [74]. Here, we did not observe any changes in receptor dependency for viruses harboring the mutation L665W. Furthermore, in a few cases L665W altered the sensitivity to other conformational antibodies suggesting it might have some effects on the structure of the E1/E2 complex. However, the substitutions at this position did not seem to affect E1/E2 interactions or cell-to-cell spread. It is conceivable that the mutations affect some other critical entry process, such as viral fusion. It will be of interest in future studies to further elucidate the role of this conserved site.

During the treatment with AR5A we observed the mutation N417D for H77/JFH1_ΔHVR1_ and I345V for J6/JFH1_ΔHVR1_. These mutations did not significantly alter AR5A susceptibility but increased viral fitness. Higher fitness of a treated virus seems to be an important predictor of whether the virus can outgrow a NAb as we previously proposed upon treatment in culture of the highly infectious HCV recombinant SA13/JFH1 [58]. The mutation N417D abolished the first N-linked glycan of the E2 protein and has been implicated both in increasing virus infectivity and in protecting the virus from NAbs [75]. Since we did not observe any change in antibody susceptibility, it is possible that the glycosylation at position N417 does not protect AR5 or even that the glycan shield is less effective in shielding HVR1-deleted viruses. The substitution N417T shifts the glycan at N417 to N415 and has been implicated in resistance against AP33, which targets the epitope at 412–423 [39]. In a separate study with another 412–423 specific antibody, HC33.1, the mutation N417T appeared at low concentrations of antibody, but was probably increasing fitness of the virus rather than conferring specific escape [41]. The mutation I345V is novel, but the near-adjacent substitution, I347L, has been identified as a compensatory mutation adapting the HVR1-deleted Jc1 virus to cell culture [46]. Thus, the two adaptive envelope mutations N417D and I345V are probably decreasing the effectiveness of AR5A in culture through increased viral fitness.

The substitution S680T was of interest as it compensated for the fitness loss of L665S in J6/JFH1 and also increased AR5A-specific resistance. However, S680T had the opposite effect on neutralization sensitivity in the absence of L665S, hinting at a complex relationship between positions 665 and 680 while also suggesting that S680 is not part of the AR5A epitope. Several studies have described mutations that can indirectly alter HCV antibody susceptibility without themselves being a part of the antibody epitope [41;51;68;69;76]. Moreover, the phenotype of some of these mutations has been shown to depend on the envelope protein context [41;68]. However, S680T appears to be unique, as it modulates AR5A sensitivity in a position-665-dependent manner and simultaneously compensates for the lower viability of viruses harboring the L665S escape mutation. Since H77/JFH1 has threonine at position 680 (as does most genotype 1, 3, 4 and 5 isolates of HCV, whereas genotype 2 and 6 typically have serine at this position) and the virus H77/JFH1_L665S_ was not viable, our results indicate that the compensatory effect of S680T for mutation L665S was specific for J6/JFH1, indicating that the two pathways to resistance (L665W for H77/JFH1 and L665S/S680T for J6/JFH1) are likely, at least partly, isolate-dependent. It is important that we observed isolate-dependent pathways requiring either one or two mutations to confer complete resistance of a viable virus as the former would be considered to have a low barrier to resistance whereas the other is relatively high. Using HVR1-deleted viruses enable researchers to perform escape studies of multiple isolates from different genotypes, providing relevant information even for otherwise resistant isolates, such as J6/JFH1.

In conclusion, using neutralization-sensitive HVR1-deleted viruses we were able to generate escape viruses in vitro against the HMAb AR5A. We showed that the selective pressure can result in different escape mutations depending on the envelope protein sequence. We identified novel escape mutations at positions either involved directly in binding, such as L665S and L665W or indirectly, such as S680T. Our data emphasizes the complexity of viral resistance against conformational antibodies, in this case HMAb AR5A, and highlights the importance of studying resistance using multiple isolates, preferably both of high and low inherent neutralization sensitivity. These important findings are only attainable through escape studies and are highly relevant in the context of viral infectivity and fitness. The knowledge gained concerning pathways and barriers to antibody resistance will aid the development of a broadly effective HCV vaccine.

## Materials and methods

### Antibodies and reagents

Human monoclonal antibodies AR3A, AR4A, and AR5A against HCV structural proteins were produced as described previously [27;33]. Antibodies against HCV receptors were anti-CD81 (BD Pharmingen Cat. JS81), anti-SR-BI C16-17 [67;77] and polyclonal anti-LDLr (R&D Systems Cat. AF2148). Control antibodies for receptor blocking assays were the isotype antibody 553447 for CD81, the antibody D for SR-BI [77], and a goat IgG (R&D Systems Cat. AB-108C) for LDLr. Soluble CD81 protein was a kind gift from Steven Foung [73]. Adapted recombinants with the core-NS2 sequence from genotypes 1a (H77), 2a (J6), 3a (S52), 4a (ED43), 5a (SA13) and 6a (HK6a) and UTR´s as well NS3-NS5B region from genotype 2a (JFH1) with or without HVR1 were described previously [44;54;58–61]. H77 E1E2 expression plasmids, previously validated for functionality in HCVpp assays [67], were used for E1/E2 interaction studies. Plasmids with point mutations were generated by conventional cloning techniques (Fusion PCR and Quikchange). The HCV sequence of the final plasmid DNA preparations were confirmed by direct sequencing (Macrogen). Antibody against NS5A, 9E10 [61], was provided by Charles Rice.

### Cell culture of Huh7.5 cells

Huh7.5 cells [78], provided by Charles Rice, were cultured in Dulbecco’s modified eagle medium DMEM (Gibco/Invitrogen Corporation, Carlsbad, CA) supplemented with 10% of heat-inactivated fetal bovine serum (FBS), penicillin 100 U/mL and streptomycin 100 μg/mL (Gibco/Invitrogen Corporation) at 5% of CO_2_ at 37°C. Cells were split every 48 to 72 hours. Virus stocks were generated by low MOI infection of naive Huh7.5 cells. Supernatants were collected at the peak of infection, then filtered and stored at -80°C. HCV envelope ORF sequencing from culture supernatants was done by long RT-nested PCR procedures, as described [44;54;58–60].

### Transfection of Huh7.5 cells

Huh7.5 cells were plated at 4x10^5^ per well in 6-well plates 24 hours prior to transfection at which point they were ~70% confluent. Plasmids were linearized by XbaI treatment (New England BioLabs). RNA was generated by T7-mediated in-vitro transcription and was transfected into Huh7.5 cells using lipofectamine 2000 (Invitrogen). 6 hours post transfection, Huh7.5 cells were trypsinized and reseeded into four wells of 24-well plates at a cell density of 8x10^4^ (for the 4 time-points of the assay) along with plating in 6-well chamber slides for assessing percent infected cells at the four time-points, using the primary mouse anti-HCV NS5A 9E10 antibody and the secondary antibody Alexa488 goat anti-mouse IgG (H+L) (Invitrogen) as described [59] and Hoechst 33342 (Molecular Probes) counterstain for nuclei. Viral spread was monitored every 24 hours along with harvesting of virus supernatant up to 96 hours post transfection. Supernatants collected during experiments were sterile filtered and stored at −80°C. The virus titers were determined as described previously [79, 80].

### HCV escape assay

Huh7.5 cells (~70% confluent) were infected with HCV virus H77/JFH1 or J6/JFH1 with or without HVR1 at an MOI of 0.001 and incubated in 5% of CO_2_ at 37°C. Virus infection was monitored by immuno-staining every 2–3 days as described above. After the virus infection spread to 1–10%, the indicated concentrations of antibody AR5A were added in each well. Supernatants were collected and filtered when cell infection reached 80–90% of the cells.

### HCV neutralization

6x10^3^ Huh7.5 cell per well was plated in poly-D-lysine 96-well plates and incubated for 24 hours. Next day, a volume of virus stock corresponding to a read-out of 50–300 focus forming units per well (FFUs/well) were incubated in quadruplicates with a dilution series of monoclonal antibody and relevant control antibody. Virus-antibody mixes along with eight replicates of virus only were incubated for 1 hour at 37°C and were added to Huh7.5 cells (~70% confluent) and incubated for 4 hours at 37°C and 5% CO_2_. Subsequently, the cells were washed and fresh medium was added prior to incubation for a total infection time of 48 hours. Cells were fixed and stained with 9E10 antibody as described previously [59]. The data was normalized to 8 replicates of virus only and analyzed using three or four parameters curve fitting in GraphPad Prism [67]. In all cases, the neutralization data were confirmed in two independent experiments.

### HCV receptor blocking

6x10^3^ Huh7.5 cells per well was plated in poly-D-lysine 96–well plates and incubated for 24 hours. The next day the Huh7.5 cells were incubated in four replicates with a 1 to 5 dilution series of monoclonal antibody against CD81 or SR-BI receptors or the polyclonal antibody against LDLr and four replicates of the respective control antibodies at the highest concentrations used in the assay. A volume of virus stock corresponding to a read-out of 50–300 FFUs/well of HCV were added to the cell-antibody mix and incubated for 4 hours at 37°C (cells were ~70% confluent at time of infection). Cells were washed and fresh medium was added prior to incubation for a total infection time of 48h. Cells were fixed and stained with 9E10 antibody as described [59]. The data was normalized to 8 replicates of virus only and analyzed using four parameters curve fitting in GraphPad Prism. Blocking data were confirmed in two independent experiments.

### Cell-to-cell spread

Huh7.5 cells were plated at 400.000 cells/well in 6-well plates and the following day the cells were transfected with HCV RNA transcripts (cells were ~70% confluent) as previously described [59]. The following day cells were trypsinized and mixed with naive trypsinized Huh7.5 cells prior to plating at 12,000 Huh7.5 cells per well in poly-D-lysine 96–well plates (NUNC). 6 wells per plate for each virus condition were seeded along with 12 wells per plate with only naive Huh7.5 cells to estimate background staining on each plate. A ratio of transfected/naive cells of 1:150 was used for cells plated in standard medium and a ratio of 1:30 was used for cells plated in standard medium in the presence of 10 μg/ml of the cross-genotype reactive HCV neutralizing antibody, AR3A [33]. This dose represented at least 500-fold the IC_50_ value for the tested HVR1-deleted viruses [48]. 6 replicates of cell mixes from each virus construct were plated in the absence or presence of AR3A for fixation at the two time-points; 24 hours and 48 hours post-plating. At 24 hours and 48 hours a plate was fixed in methanol and all plates were subsequently stained for infection using the NS5A-specific antibody, 9E10. Number of single infected cells, number of focus forming units (clusters of single cells) and size of FFUs were counted and calculated using adapted BioSpot software (Cellular Technology Lmtd.). Data was analyzed using GraphPad Prism.

### E1/E2 interaction assay

293T cells (American Type Culture Collection, ID: CRL-1573) were plated at 400.000 cells/well in a 6-well plate and the following day the cells were transfected using Lipofectamine 2000 (Invitrogen) with 5 μg of E1E2 expression plasmids. The following day the cells were replated back into the 6-well plate except for the plating of two slides. The next day the slides were fixed with 4% paraformaldehyde at room temperature for 15 minutes and the cells were permeabilized with 0.1% Tween20. Subsequently the slides were stained for E2 or E1/E2 using AR3A or AR4A, respectively, followed by incubation with anti-human Alexa488 coupled secondary antibody and Hoechst 33342 (Molecular Probes) counterstain for nuclei. Transfection efficiencies were comparable. Cells in 6-wells were lysed using a 1% nDDM detergent in NativePAGE sample buffer (Novex life technologies/Thermo Scientific) supplemented with 1x Halt protease inhibitor cocktail (Thermo Scientific) by pipetting the solution up and down several times. The lysates were cleared by centrifugation at 4°C at 20.000xrcf for 30 minutes. The samples were transferred to fresh tubes, MgCl_2_ was added in a final concentration of 2mM and samples were treated with Benzonase endonuclease (Sigma) for 30 min prior to immunoprecipitation with the conformationally-reactive E2 antibody, AR3A, using Protein G coupled Dynabeads (Thermo Scientific) as per the manufacturer´s instructions. The E1/E2 protein complexes were eluted directly into LDS buffer by heating the beads for 10 minutes at 70°C. The E1/E2 protein complexes were loaded onto SDS-PAGE gels and run for 1 hour at 200 V under reducing conditions. This was followed by transfer onto a PVDF membrane at 35 V for 1 hour. E2 protein was visualized using mouse H52 antibody [81] and E1 protein was visualized on separate membranes using mouse A4 antibody [82] incubated overnight at 4°C on a shaker followed by incubation with anti-mouse coupled with horse radish peroxidase for 1 hour at room temperature on a shaker. The Supersignal West Femto Chemi-luminescence (Thermo Scientific) kit was used. The size of proteins was estimated by using the Precision Plus Protein WesternC standard (Bio-Rad) size marker as per the manufacturer´s instructions.

### Soluble CD81 neutralization

We plated 6x10^3^ Huh 7.5 cell per well in poly-D-lysine 96-well plates and incubated for 24 hours. Next day, a volume of virus stock corresponding to a read-out of 50–300 FFUs/well of HCV was incubated in four replicates with a dilution series of soluble CD81 receptor protein. Virus-CD81 mixes along with eight replicates of virus only were incubated for 1 hour at 37°C and were added to Huh7.5 cells (~70% confluent) and incubated for 4 hours at 37°C and 5% CO_2_. Subsequently, the cells were washed and incubated for a total infection time of 48h with fresh medium. Cells were fixed and stained with the 9E10 antibody as described [59]. The data was normalized to 8 replicates of virus only and analyzed using four parameters curve fitting in GraphPad Prism. Experiments data were confirmed in two independent experiments.

### Immuno-staining with antibodies AR4A and AR5A

Huh7.5 cells were infected with viruses H77/JFH1 or H77/JFH1_L665W_ with a multiplicity of infection (MOI) equal to 0.01 and incubated for 24 hours. We plated 2.5x10^4^ infected Huh7.5 cells per well in an 8 wells slide and incubated them for an additional 24 hours. Cells were fixed with para-formaldehyde at room temperature for 15 minutes and the cells were permeabilized with 0.1% Tween20. Antibody anti-NS5A in combination with either AR4A or AR5A was used as primary antibodies. We used a combination of Alexa594 goat anti-mouse IgG (H+L) (Invitrogen) and Alexa488 goat anti-human (Invitrogen) as secondary antibodies with a Hoechst 33342 (Molecular Probes) counterstain for nuclei.

### HCV RNA immunoprecipitation

Immunoprecipitation was carried out using the immunoprecipitation kit Dynabeads Protein G (Thermo Scientific) as previously described [67]. We used 5 μg of antibodies AR3A, AR4A or AR5A along with a relevant isotype antibody control b6. Magnetic bead-associated HCV RNA was eluted with lysis buffer from the QIAmp MinElute Vacuum kit (Qiagen) and the HCV RNA was extracted using the kit protocol together with a dilution series of a standard sample with a known HCV RNA concentration and a blank solution. HCV RNA was eluted in 30μl of elution buffer and 8μl of each sample was used for reverse transcription-quantitative PCR (RT-qPCR) using a Light Cycler as described before [59;67].

## Supporting information

S1 Fig Substitution L665W conferred resistance in the cultured-adapted H77/JFH1_ΔHVR1_ virus. 1st passages of the indicated viruses were subjected to a ten-fold dilution series of AR5A starting at 50 μg/ml. The virus/antibody mixes along with virus only were added to Huh7.5 cells and after 48 hour infection the cells were immunostained and the number of FFUs per well were counted as described in Materials and Methods. Neutralization data are shown as the mean of four replicates with standard error of the mean and normalized to eight replicates of virus only. Three-parameter curve-fitting was used to obtain sigmoidal dose-response curves. Error bars represent standard errors of the mean.
(TIF)

S2 Fig L665W did not generate resistance against the HMAb AR3A or AR4A in HVR1-deleted H77/JFH1 virus. 1st passages of the indicated viruses were subjected to a ten-fold dilution series of antibodies (A) AR3A or (B) AR4A starting at 50 μg/ml. The virus/antibody mixes along with virus only were added to Huh7.5 cells and after 48 hour post infection the cells were immunostained and the number of FFUs per well were counted. Neutralization data are shown as the mean of four replicates with standard error of the mean and normalized to eight replicates of virus only. Three-parameter curve-fitting was used to obtain sigmoidal dose-response curves. Error bars represent standard errors of the mean.
(TIF)

S3 Fig The HMAb AR4A binding to cells infected with virus H77/JFH1 was not affected by the substitution L665W. Huh7.5 cells were infected with virus H77/JFH1 or H77/JFH1_L665W_ were immune stained with primary antibodies against NS5A (9E10) and E1/E2 (AR4A), and specific secondary antibodies coupled to fluorophores Alexa488 or Alexa594. Nuclei were stained using Hoechst.
(TIF)

S4 Fig L665W did not affect virus inhibition by soluble CD81 neutralization. Viruses H77/JFH1 and H77/JFH1_L665W_ were subjected to a five-fold dilution series of soluble CD81 receptor starting at 50 μg/ml. The virus/antibody mixtures along with virus only were added to Huh7.5 cells and after 48 hour post infection the cells were immunostained and the number of FFUs per well were counted as described in Materials and Methods. Neutralization data are shown as the mean of four replicates with the standard error of the mean. Neutralization was related to eight replicates of virus only. Three-parameter curve-fitting was used to obtain sigmoidal dose-response curves. Error bars represent standard errors of the mean.
(TIF)

S5 Fig Cell-to-cell spread of HCV was not specifically affected by the AR5A-specific escape substitutions L665W, L665S and S680T. Huh7.5 cells were transfected with the indicated viral constructs including JFH1_ΔE1E2_ as a negative control. These were mixed with naive cells and plated at 12,000 cells/well in 96 well plates to ensure nearly 100% cell confluence. Transfected cells were diluted 1:150 with naive Huh7.5 cells for wells left untreated with neutralizing AR3A antibody and 1:30 for wells treated with AR3A. Number of FFUs, size of FFUs and number of single infected cells were counted for the 6 replicates of each virus condition using automated BioSpot software (Cellular Technology Lmtd.) following HCV-specific staining after 24 hour and 48 hour. Error bars indicate SD. (A) Average number of FFUs per well for wells not treated with AR3A antibody. (B) Average number of FFUs per well for wells treated with AR3A antibody. (C) Average size of FFUs in wells treated with AR3A. (D) Number of single infected cells per FFU in wells treated with AR3A.
(TIF)

S6 Fig The substitution A349D increased infectivity of H77/JFH1 whereas R408K had no effect on infectivity of J6/JFH1. Huh7.5 cells were transfected with in vitro transcribed RNA of the indicated (A) H77/JFH1 or (B) J6/JFH1 recombinants. Supernatants were collected and HCV infectivity titers were determined as indicated in Materials and Methods.
(TIF)

S7 Fig AR3A neutralization of HCV prototype isolates of genotypes 1–6 with and without L665W. 1st passage virus stocks of the indicated viruses were subjected to dilution series of AR3A. The virus/antibody mixes along with virus only were added to Huh7.5 cells and after 48 hour infection the cells were immunostained and the number of FFUs per well were counted. Values are means of four replicates and normalized to 8 replicates of virus only. Three-parameter or four-parameter curve-fitting was used to obtain sigmoidal dose-response curves. Errors bars represent the standard errors of the mean. *Virus harbored the substitution I262L. ^†^Virus harbored the substitution I355F. ^‡^Virus harboring substitutions A372V, Q454H and F580V.
(TIF)

S8 Fig AR4A neutralization of HCV prototype isolates of genotypes 1–6 with and without L665W. 1st passages of the indicated viruses were subjected to dilution series of AR4A. The virus/antibody mixes along with virus only were added to Huh7.5 cells and after 48 hour post infection the cells were immunostained and the number of FFUs per well were counted. Values are means of four replicates and normalized to 8 replicates of virus only. Three-parameter or four-parameter curve-fitting was used to obtain sigmoidal dose-response curves. Errors bars represent the standard errors of the mean. *Virus harbored the substitution I262L. ^†^Virus harbored the substitution I355F. ^‡^Virus harboring substitutions A372V, Q454H and F580V.
(TIF)
