# Supplementary figures and images for: Applying antibody-sensitive hypervariable region 1-deleted hepatitis C virus to the study of escape pathways of neutralizing human monoclonal antibody AR5A
Source: PLoS Pathog. 2017 Feb 23;13(2):e1006214. doi: 10.1371/journal.ppat.1006214 (PMC5358973; doi:10.1371/journal.ppat.1006214)

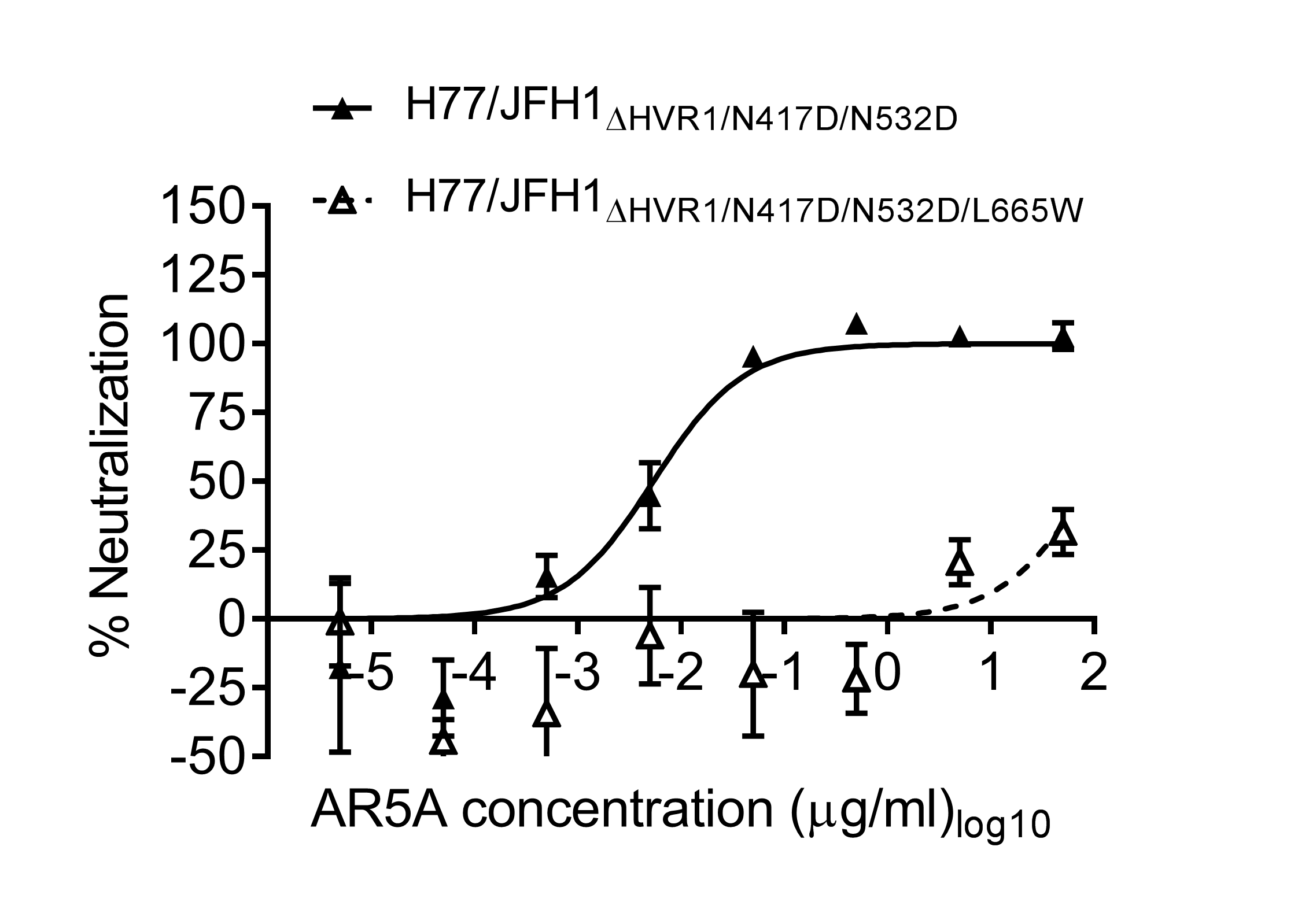

Supplement: S1 Fig — 1st passages of the indicated viruses were subjected to a ten-fold dilution series of AR5A starting at 50 μg/ml. The virus/antibody mixes along with virus only were added to Huh7.5 cells and after 48 hour infection the cells were immunostained and the number of FFUs per well were counted as described in Materials and Methods. Neutralization data are shown as the mean of four replicates with standard error of the mean and normalized to eight replicates of virus only. Three-parameter curve-fitting was used to obtain sigmoidal dose-response curves. Error bars represent standard errors of the mean. (TIF) [file ppat.1006214.s001.tif]

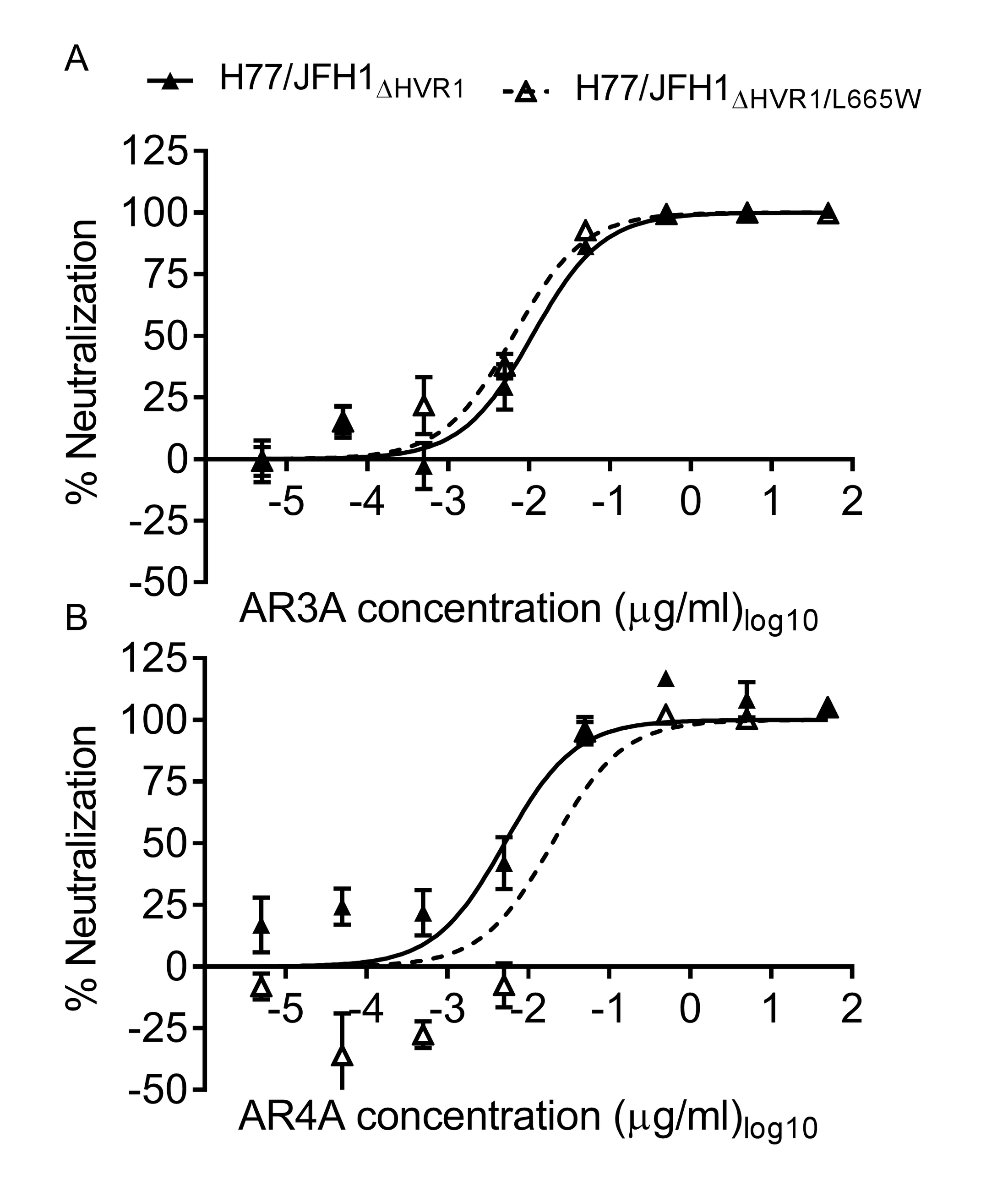

Supplement: S2 Fig — 1st passages of the indicated viruses were subjected to a ten-fold dilution series of antibodies (A) AR3A or (B) AR4A starting at 50 μg/ml. The virus/antibody mixes along with virus only were added to Huh7.5 cells and after 48 hour post infection the cells were immunostained and the number of FFUs per well were counted. Neutralization data are shown as the mean of four replicates with standard error of the mean and normalized to eight replicates of virus only. Three-parameter curve-fitting was used to obtain sigmoidal dose-response curves. Error bars represent standard errors of the mean. (TIF) [file ppat.1006214.s002.tif]

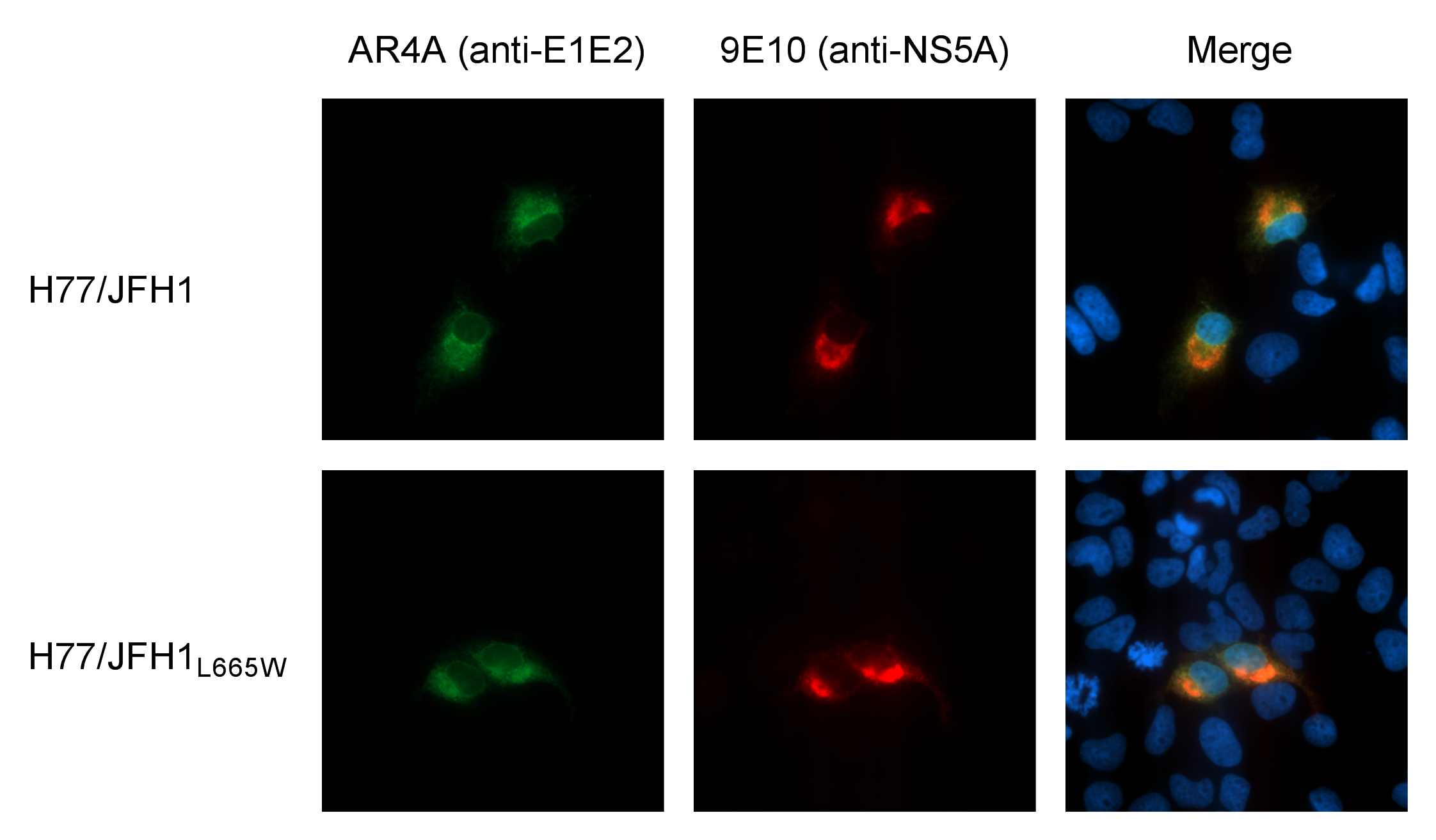

Supplement: S3 Fig — Huh7.5 cells were infected with virus H77/JFH1 or H77/JFH1L665W were immune stained with primary antibodies against NS5A (9E10) and E1/E2 (AR4A), and specific secondary antibodies coupled to fluorophores Alexa488 or Alexa594. Nuclei were stained using Hoechst. (TIF) [file ppat.1006214.s003.tif]

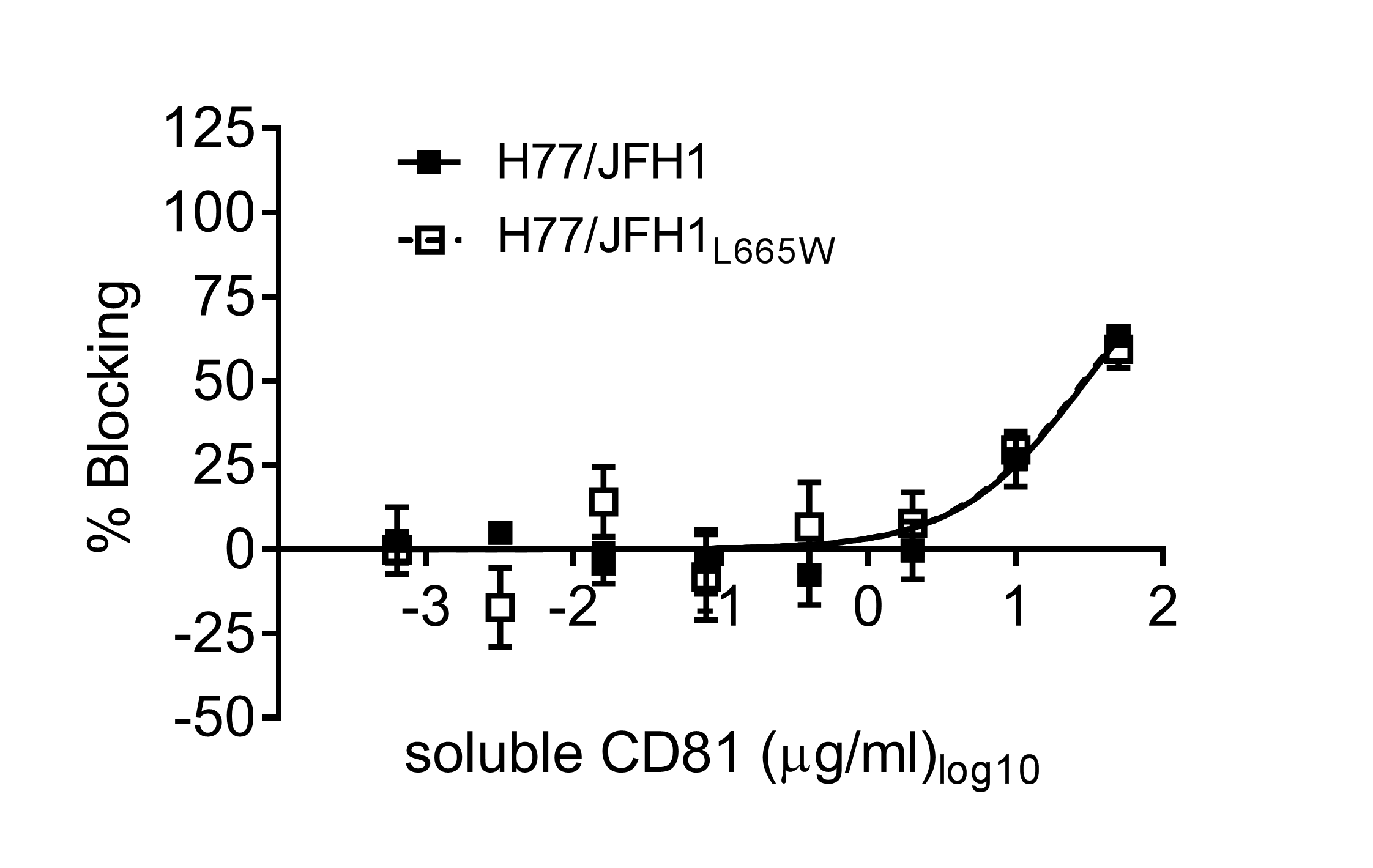

Supplement: S4 Fig — Viruses H77/JFH1 and H77/JFH1L665W were subjected to a five-fold dilution series of soluble CD81 receptor starting at 50 μg/ml. The virus/antibody mixtures along with virus only were added to Huh7.5 cells and after 48 hour post infection the cells were immunostained and the number of FFUs per well were counted as described in Materials and Methods. Neutralization data are shown as the mean of four replicates with the standard error of the mean. Neutralization was related to eight replicates of virus only. Three-parameter curve-fitting was used to obtain sigmoidal dose-response curves. Error bars represent standard errors of the mean. (TIF) [file ppat.1006214.s004.tif]

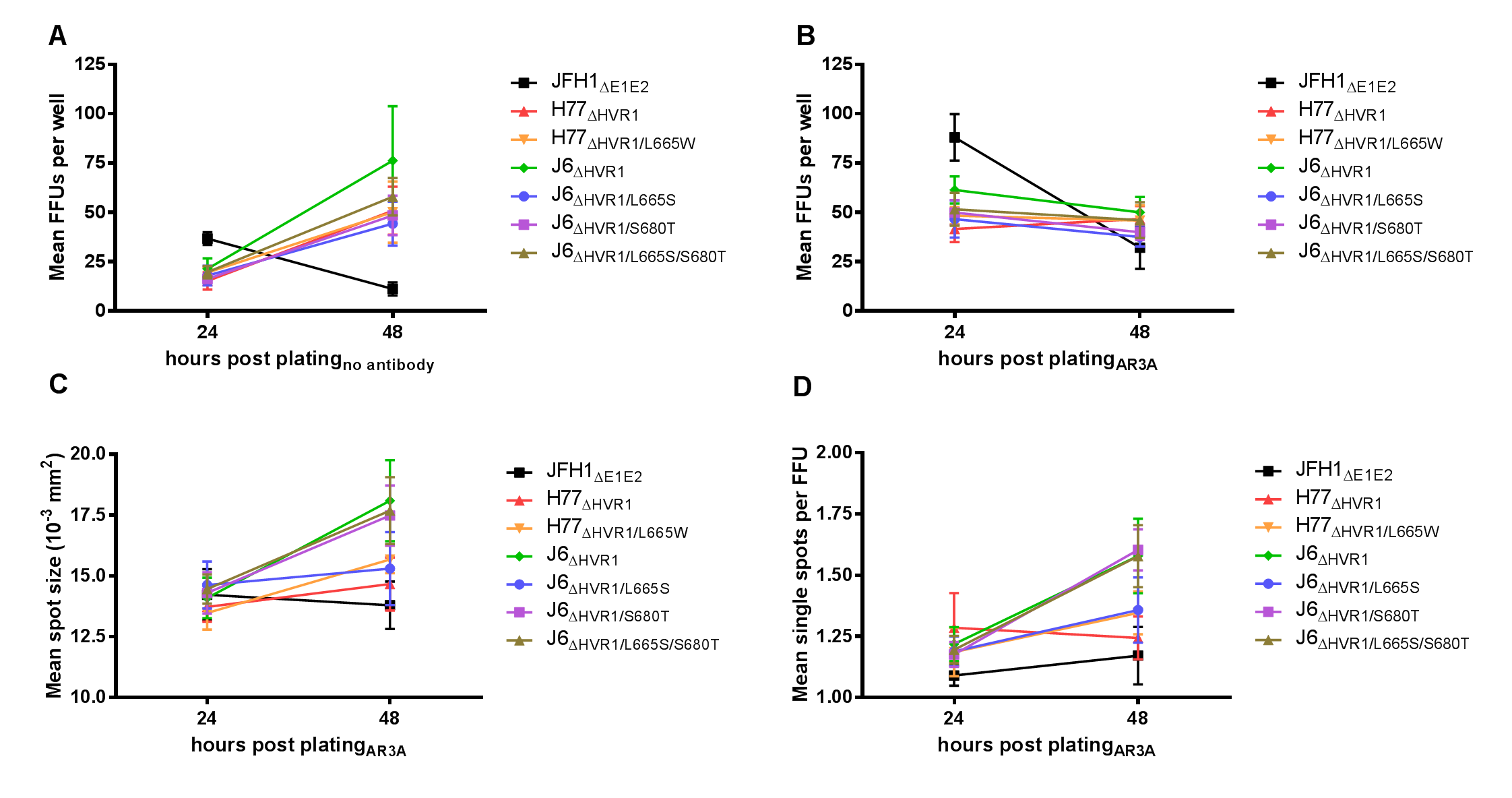

Supplement: S5 Fig — Huh7.5 cells were transfected with the indicated viral constructs including JFH1ΔE1E2 as a negative control. These were mixed with naive cells and plated at 12,000 cells/well in 96 well plates to ensure nearly 100% cell confluence. Transfected cells were diluted 1:150 with naive Huh7.5 cells for wells left untreated with neutralizing AR3A antibody and 1:30 for wells treated with AR3A. Number of FFUs, size of FFUs and number of single infected cells were counted for the 6 replicates of each virus condition using automated BioSpot software (Cellular Technology Lmtd.) following HCV-specific staining after 24 hour and 48 hour. Error bars indicate SD. (A) Average number of FFUs per well for wells not treated with AR3A antibody. (B) Average number of FFUs per well for wells treated with AR3A antibody. (C) Average size of FFUs in wells treated with AR3A. (D) Number of single infected cells per FFU in wells treated with AR3A. (TIF) [file ppat.1006214.s005.tif]

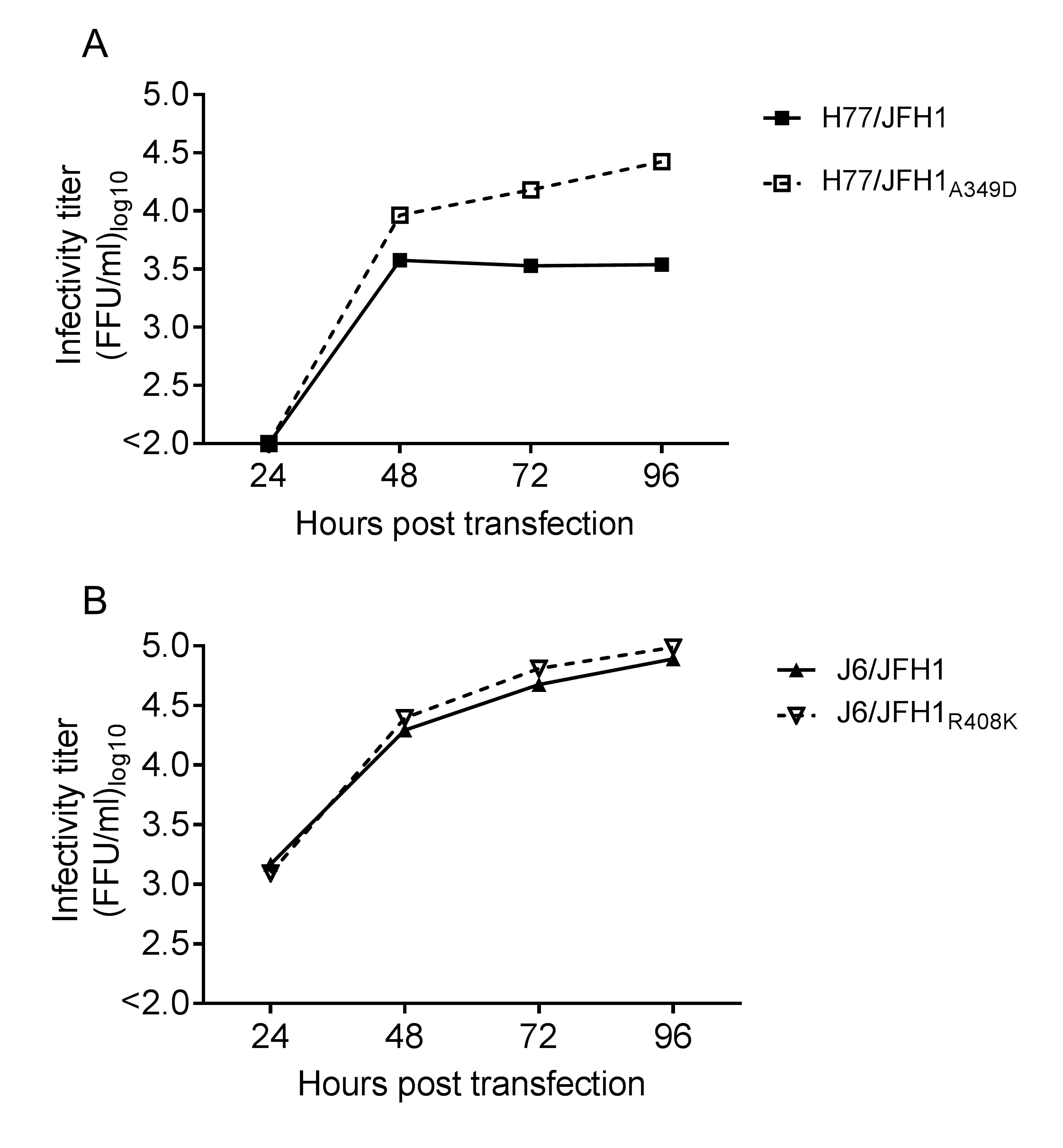

Supplement: S6 Fig — Huh7.5 cells were transfected with in vitro transcribed RNA of the indicated (A) H77/JFH1 or (B) J6/JFH1 recombinants. Supernatants were collected and HCV infectivity titers were determined as indicated in Materials and Methods. (TIF) [file ppat.1006214.s006.tif]

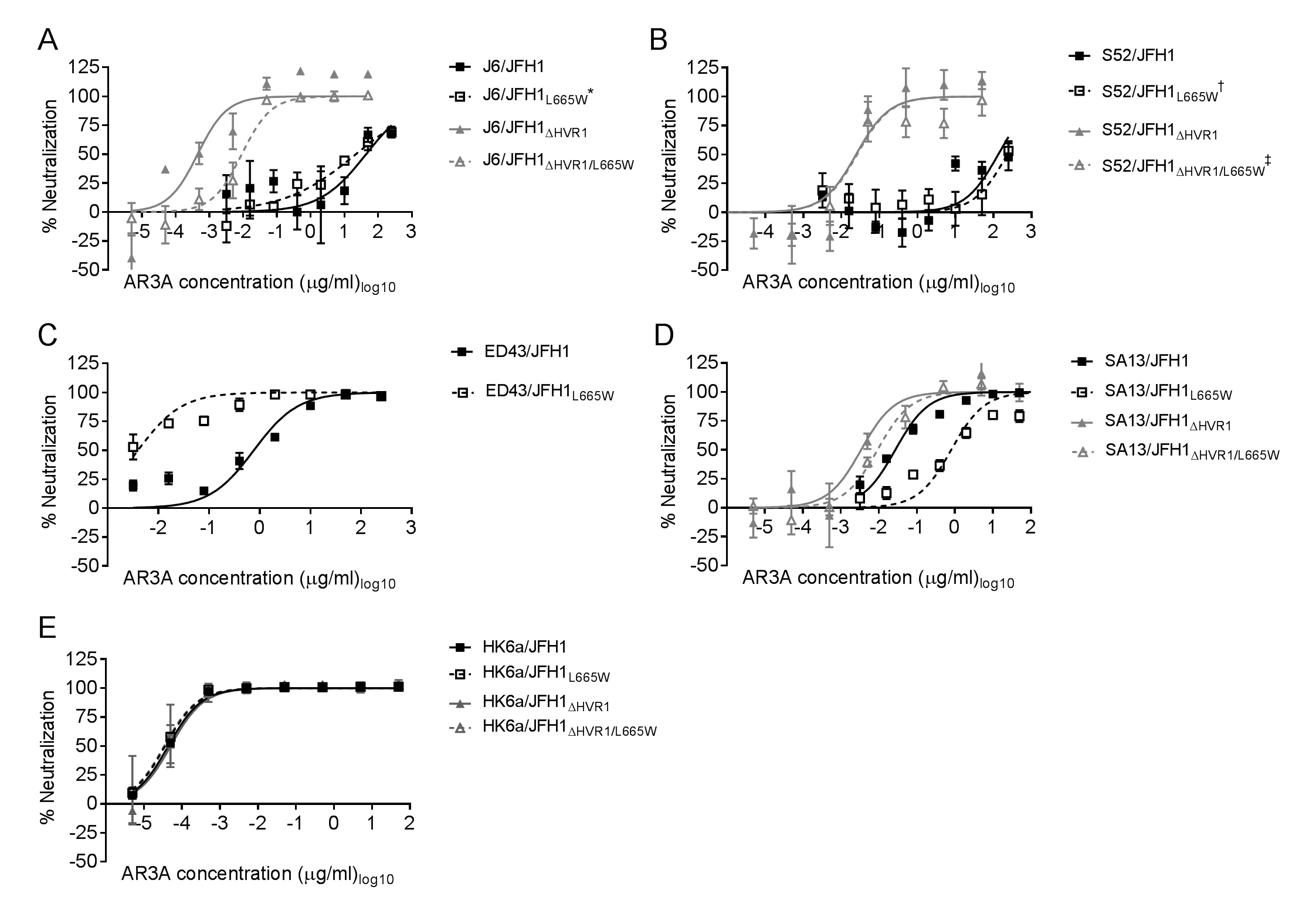

Supplement: S7 Fig — 1st passage virus stocks of the indicated viruses were subjected to dilution series of AR3A. The virus/antibody mixes along with virus only were added to Huh7.5 cells and after 48 hour infection the cells were immunostained and the number of FFUs per well were counted. Values are means of four replicates and normalized to 8 replicates of virus only. Three-parameter or four-parameter curve-fitting was used to obtain sigmoidal dose-response curves. Errors bars represent the standard errors of the mean. *Virus harbored the substitution I262L. †Virus harbored the substitution I355F. ‡Virus harboring substitutions A372V, Q454H and F580V. (TIF) [file ppat.1006214.s007.tif]

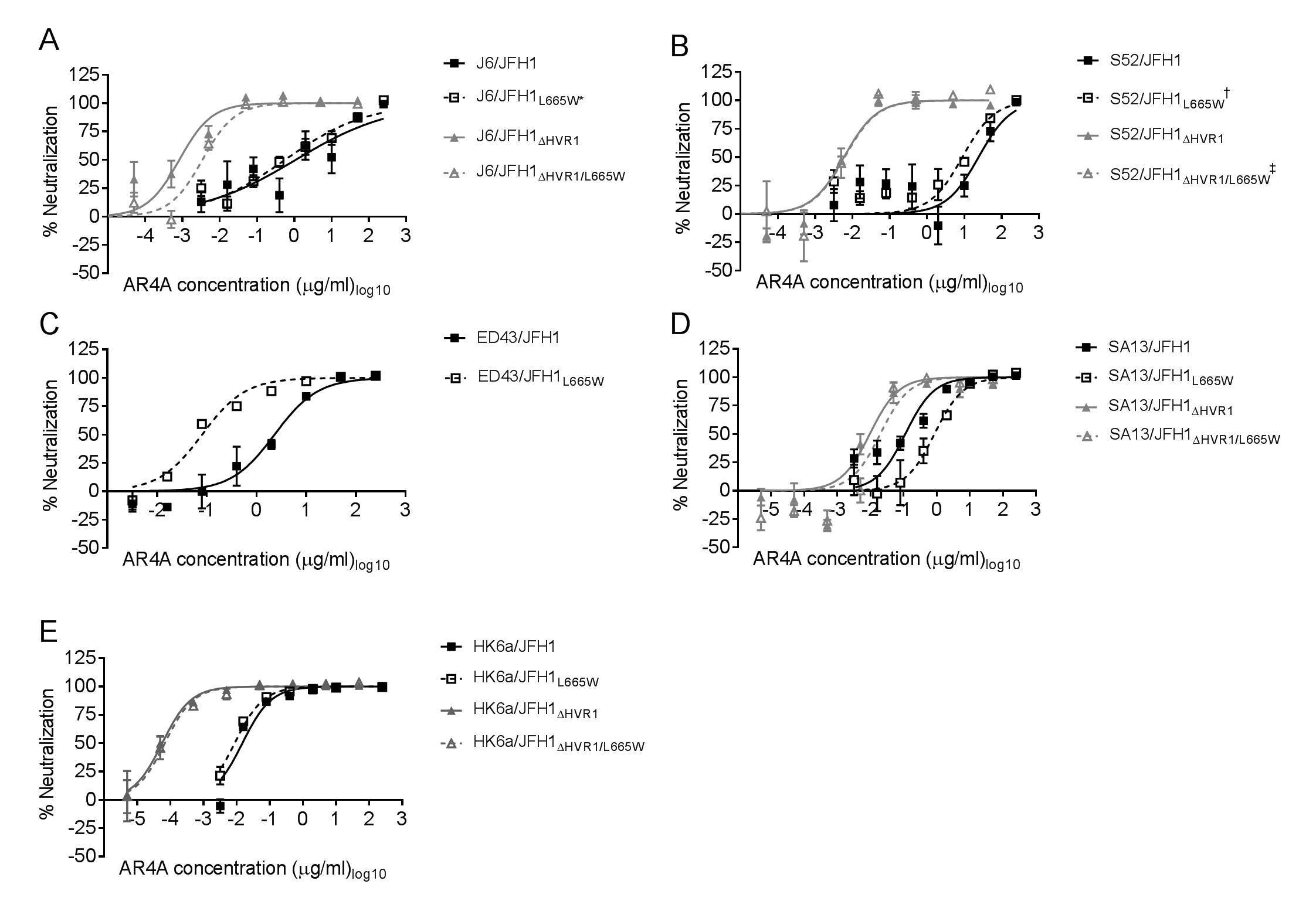

Supplement: S8 Fig — 1st passages of the indicated viruses were subjected to dilution series of AR4A. The virus/antibody mixes along with virus only were added to Huh7.5 cells and after 48 hour post infection the cells were immunostained and the number of FFUs per well were counted. Values are means of four replicates and normalized to 8 replicates of virus only. Three-parameter or four-parameter curve-fitting was used to obtain sigmoidal dose-response curves. Errors bars represent the standard errors of the mean. *Virus harbored the substitution I262L. †Virus harbored the substitution I355F. ‡Virus harboring substitutions A372V, Q454H and F580V. (TIF) [file ppat.1006214.s008.tif]
